# Linear Actuation of Dielectrophoretic Formed Multi-Walled Carbon Nanotube Fiber with Carbide-Derived Carbon in Polar Aprotic and Polar Protic Solvents

**DOI:** 10.3390/ma18143254

**Published:** 2025-07-10

**Authors:** Chau B. Tran, Quoc Bao Le, Rudolf Kiefer

**Affiliations:** 1 Faculty of Applied Sciences, Ton Duc Thang University, Ho Chi Minh City 700000, Vietnam; tranboichau@tdtu.edu.vn; 2 Conducting Polymers in Composites and Applications Research Group, Faculty of Applied Sciences, Ton Duc Thang University, Ho Chi Minh City 700000, Vietnam; qle@pittstate.edu; 3 National Institute for Materials Advancement, Pittsburg State University, Pittsburg, KS 66762, USA

**Keywords:** CNT, CNTCDC, fiber actuator, PC and aq solvent, supercapacitors

## Abstract

Carbon nanotube (CNT) fiber research focuses on developing functional fabrics with dual or multifunctional capabilities. This study investigates CNT fibers fabricated via dielectrophoresis (DEP) with the incorporation of 10 wt.% carbide-derived carbon (CDC), referred to as CNTCDC fibers. The linear actuation behavior of the CNT and the CNTCDC fibers is compared using identical electrolyte concentrations in both a polar aprotic solvent (propylene carbonate, PC) and a polar protic solvent (aqueous solution, aq). Electromechanical deformation (EMD) is studied through cyclic voltammetry and chronoamperometry. The CNTCDC fiber outperformed the pristine CNT fiber, exhibiting primary expansion during discharge in PC (stress: 1.64 kPa, strain: 0.1%) and during charge in water (stress: 1.32 kPa, strain: 0.047%). By contrast, the pristine CNT fibers showed mixed actuation responses in both solvents, resulting in diminished net stress and strain. Chronopotentiometric measurements indicated that the CNTCDC fibers achieved their highest specific capacitance in aqueous media, reaching 223 ± 17 F g^−1^ at ±0.8 A g^−1^, with a capacity retention of 94.2% at ±32 A g^−1^. Fundamental characterization techniques, including scanning electron microcopy (SEM), energy-dispersive X-ray spectroscopy (EDX), and Raman spectroscopy, are employed to analyze fiber morphology and composition. The dual functionality of CNTCDC fibers, as both actuators and energy storage elements, is demonstrated.

## 1. Introduction

Fiber-based carbon nanotube (CNT) composites, whether composed of multi-walled (MWCNT) or single-walled (SWCNT) nanotubes, are at the forefront of research due to their multifunctional applications in smart textiles [1], actuators [2], sensors [3], and energy storage devices [4]. Owing to their high strength and flexibility, CNT fibers have been proposed for use in protective gear, such as bulletproof vests [5], and as reinforcement materials in structural composites. Their exceptional electrical and thermal conductivity has also made them ideal candidates for innovative electronic applications [6]. Various fabrication methods exist for producing pristine CNT fibers or yarns [7,8]. One common approach involves drawing fibers from brush-like CNT arrays [9], or creating metal core-spun CNT yarns in linear or twisted forms, especially relevant for their application in textile-integrated supercapacitors [10]. Wet spinning of MWCNTs has also been widely adopted, producing highly conductive fibers suitable for use as electrical cables or sensors [11].

This study focused on CNT (multi-walled) and carbide-derived carbon (CDC) composite fibers, with particular emphasis on their dual function as electrochemical actuators and supercapacitors. Our fabrication approach differs from the aforementioned methods. We employed DEP assembly to form CNT fibers [12], a process that uses an aqueous suspension of SWCNT [13] or MWCNT [14], with an alternating current (AC) voltage applied between a fine needle and a substrate. Previous studies have explored the feasibility of forming such fibers in aqueous or organic media [15] and the optimal conditions for obtaining aligned MWCNT fibers [16]. The resulting fiber diameters range from 1 μm to 1 mm, with van der Waals forces enabling the cohesion of the highly porous CNT structures. Control over fiber diameter can be achieved through adjustment of the applied voltage and drawing speed [14]. While most prior research has concentrated on enhancing mechanical strength, electrical conductivity, and supercapacitor performance, most actuator-focused studies have primarily targeted CNT yarns [17,18].

Previous research conducted by our group demonstrated that CDC particles [19] can be directly incorporated into DEP formed CNT fibers [20], with CDC loadings reaching up to 75 wt.% [21]. Studies have already been performed on CNT fibers containing 25%, 50%, and 75% CDC by weight. It was observed that increasing CDC content enhances the linear actuation response in the polar aprotic solvent, such as propylene carbonate (PC), where the mixed linear actuation behavior becomes more prominent. The actuation mechanism in carbon-based materials, including CNTs and CDC, occurs within the electrolytes primarily followed by a non-faradaic process [22]. It involved the formation of an electrical double layer (EDL) on the charged carbon surfaces, which induced changes in the length of the C–C bonds and led to dimensional deformation [23]. Various studies have explored the bending actuators based on CDC [24], where the pore size is crucial [25]. Typically, CDC-based electrodes require a binder, such as PVdF-HFP (poly(vinylidene fluoride-co-hexafluoropropylene)), to maintain structural integrity. CDC materials follow an EDL-driven actuation mechanism [22]. However, some studies have reported mixed-ion actuation behavior [26], attributed to the ingress of ions into the microporous structure of the CDC and the subsequent EDL formation during discharge (with cations contributing to the EDL).

Recent work on fiber-based composites fabricated via extrusion, comprising MWCNTs and cellulose, has shown that the actuation direction can reverse depending on whether a polar aprotic or a polar protic solvent is used [27]. Building upon these observations, the present work aims to investigate the electromechanical deformation (EMD) behavior of novel DEP-formed CNT fibers containing 10 wt.% CDC (referred to as CNTCDC fibers). The linear actuation properties were examined in both the polar aprotic (propylene carbonate, PC) and the polar protic (aqueous, aq) solvents using a consistent electrolyte (LiTFSI). Additionally, the electrochemical capacitance and energy storage performance of these fibers are evaluated.

EMD measurements of the CNT and the CNTCDC fibers were performed using various electrochemical techniques. Cyclic voltammetry (scan rate 5 mV s^−1^) and chronoamperometry (frequency range: 0.0025 Hz to 0.1 Hz) were carried out within an applied potential window of 0.65 V to −0.6 V. Chronopotentiometry was used to determine the specific capacitance of each fiber type. Morphological and compositional characterization was conducted using SEM with cross-sectional imaging, Raman spectroscopy, and energy-dispersive X-ray (EDX) analysis. The Young’s modulus of the fibers—measured in dry conditions, in both the polar aprotic and the polar protic solvents containing electrolytes—was determined from stress–strain experiments. For both fiber types, a minimum of three independent samples were tested. The results are reported as mean values with corresponding standard deviations.

## 2. Materials and Methods

### 2.1. Materials

The CDC material was purchased from Skeleton Technology Ltd. (Tallinn, Estonia) and used without further purification. The material characterization using BET measurements of CDC-TiC-800 had a surface area of 1470 m^2^ g^−1^, an average particle size of 1–3 µm, a micropore volume of 0.57 cm^3^ g^−1,^, and an average pore size of 1.02 nm. The MWCNTs were purchased from Sigma-Aldrich (Taufkirchen, Germany) with material characterization from the supplier having a BET surface area of 40–300 m^2^ g^−1^, an average pore size distribution in a broad range between 7 and 15 nm (inner diameter of 3–6 nm with a length of 0.5 to 200 µm). Polyvinylpyrrolidone (PVP, mol. wt.% 40.000), bis(trifluoromethanesulfoni)mide lithium salt (LiTFSI, 99.95%), propylene carbonate (PC, 99%), and Milli-Q+ water were obtained from Sigma-Aldrich (Taufkirchen, Germany) and used as supplied.

### 2.2. CNT and CNTCDC Fiber Generation

The DEP-formed CNT fiber [13] was fabricated from an aqueous solution of PVP, MWCNTs (CNT), and Milli-Q+ water in a weight ratio of 1:4:1500, respectively. The solution was sonicated in an ice bath using an ultrasonicator (Hielscher UPS200S, Teltow, Germany) at 50% amplitude for 30 min. The PVP serves as a surfactant to prevent CNT agglomeration in the solution. For the CNTCDC fibers, 10 wt.% CDC was added to the mixture, yielding a composition (wt.%) of 1:0.5:3.5:1500 for PVP, CDC, CNT, and Milli-Q+, respectively. The general procedure for the DEP fiber formation followed the previously published methods in [28], with modifications for CDC incorporation as described in [20]. The resulting fiber lengths ranged from 1 to 5 cm. Compared to the CNT fibers drawn from the CNT forests, the DEP-formed CNT and CNTCDC fibers were typically more brittle. Fiber diameters, determined from SEM images, were 149 ± 11 µm for the CNT fibers and increased slightly to 153 ± 12 µm with the addition of CDC. Fiber mass was measured using an analytical balance (Mettler Toledo, Columbus, OH, USA; readability: 0.002 mg to 0.1 mg). For a fiber length of 1.1 cm, the average mass was 58.6 ± 4.8 µg for the CNT fibers and 62.8 ± 5.2 µg for the CNTCDC fibers. The corresponding densities were calculated as 0.30 ± 0.02 g cm^−3^ and 0.31 ± 0.02 g cm^−3^_,_ respectively.

### 2.3. Electromechanical Deformation

EMD measurements of the CNT and CNTCDC fibers were performed using a custom-built (Linear actuation stage, LAS, Physik instrumente M-414.3PD, min step size 0.5 µm, Karlsruhe, Germany) linear muscle analyzer [29]. The CNT or CNTCDC fiber was fixed at one end on the force sensor (TRI202PAD, Panlab, Barcelona, Spain) with clamps of a size of 5 mm length and on the other side of a solid arm with gold contacts. The fiber was fully immersed in an electrolyte consisting of 0.1 M LiTFSI, either in an aqueous (aq) or a propylene carbonate (PC) solution. The gold-contact arm functioned as the working electrode, with a platinum sheet (9 cm^2^) as the counter electrode and an Ag/AgCl (3 M KCl) electrode as the reference. All electrodes were connected to a potentiostat (Biologic PG581, Seyssinet-Pariset, France) operated via custom in-house software. The electrochemical measurements were synchronized in real-time with force data, allowing stress calculation (σ = F/A, where F is force and A is cross-sectional area) under isometric EMD conditions at a fixed fiber length of 1 mm.

The force sensor of the linear muscle analyzer can only measure the mass change with the calibration of mass/µm and will give the k factor to translate the mass change in the length change (calculated to strain ε (%) = Δl/l × 100) as isotonic EMD (constant mass of 50 mg equal 0.5 mN). The linear muscle analyzer includes a movable stage, and allows for individual calibration of each fiber based on its Young’s modulus. This calibration (k factor) is more specific than commercial linear muscle analyzers, and enables the modulus determination prior to each measurement. The k factor also allows for assessing potential changes in fiber properties post-actuation.

Cyclic voltammetry (scan rate 5 mV s^−1^) was conducted simultaneously with measurements of mass (stress) and length (strain) changes over a potential range from 0.65 V to 0.6 V in both the LiTFSI-aq and the LiTFSI-PC electrolytes. Chronoamperometric experiments were performed at frequencies ranging from 0.0025 Hz to 0.1 Hz. Integration of current density–time curves at each frequency enabled the stress and strain behavior analysis in relation to the charge density.

Chronopotentiometric measurements of the CNT fiber at varied current densities (current i/ mass m) ± 0.85 A g^−1^, ± 1.7 A g^−1^, ± 3.4 A g^−1^, ± 8.5 A g^−1^, ± 17 A g^−1^, and ± 34 A g^−1^, having a constant charge density of ± 180 C g^−1^. The CNTCDC fiber current density i/m is slightly lower with ± 0.8 A g^−1^, ± 1.6 A g^−1^, ± 3.2 A g^−1^, ± 8.0 A g^−1^, ± 16 A g^−1^, and ± 32 A g^−1^ having a constant charge density of ± 160 C g^−1^. The specific capacitance C_s_ is obtained from Equation (1) [30].

(1) $$C_{s}=\frac{i}{-slope\cdot m}$$

The slope is obtained at each chronopotentiogram for each current density i/m of the potential time curve (after IR drop). From each CNT and CNTCDC fiber, three independent fibers were produced and measured to obtain the mean values with standard deviation, ensuring reproducibility.

### 2.4. Characterizations

The surface and cross-section (broken under liquid nitrogen) images of the CNT and CNTCDC fibers were made using SEM (Tescan Orsay Holding, Brno-Kohoutovice, Czech Republic). Raman spectroscopy (Renishaw plc, resolution 2 cm^−1^, Wotton-under-Edge, UK) of the CNT and CNTCDC fibers was performed using a 514 nm Argon laser at Raman shifts between 1800 cm^−1^ and 1200 cm^−1^. EDX spectroscopy (EDX, Oxford Instruments with X-Max 50 mm^2^ detector, High Wycombe, UK) of the fiber was made directly after the actuation measurements at charging (3 min, 0.65 V), with a small piece broken, dried at 2 mbar and 80 °C, and EDX measurements of the section image performed. The rest of the fiber was discharged (−0.6 V, 3 min) and obtained using the same procedure as the former, with EDX spectroscopy of the cross-section performed. The CNT and CNTCDC fibers were brittle and, to assure reliable resistivity measurements, graphite conductive adhesive (Electron Microscopy Sciences, Hatfield, PA, USA) was placed on both ends of the fiber. To obtain the resistivity of the fiber, a two-point probe was applied using a digital multimeter (LCR200 Meter, EXTECH instruments, Nashua, NH, USA) to calculate the electronic conductivity σ_e_ (Equation (2)) [31].

(2) $$\sigma_{e}=\frac{l}{R\cdot A}$$

The length l of the fiber (between contacts, in general, 1 mm was used) with the obtained resistivity R and the cross-section area A (cylindrical fiber, A = 2·π·r·l + 2·π·r^2^, with r as radius and l as the length of the fiber). From each fiber sample (CNT or CNTCDC), at least 5 were measured, with the conductivity results presented in mean values with standard deviations.

The CNT and CNTCDC fiber were subjected to strain–stress measurements to obtain the Young’s modulus, and were made using the same linear muscle setup. The fiber probes were fixed between the force sensor and static arm, and then the movable stage was used to stretch the fiber (limit 500 nm) in a given program. The same measurements were performed in the electrolyte LiTFSI with the solvents PC and aq.

## 3. Results and Discussion

Previous studies have demonstrated that DEP-formed CNT fibers exhibit linear actuation behavior [20], particularly in LiTFSI-PC [21], though often with mixed actuation responses during the charging and discharging cycles. Incorporating the CDC up to 75 wt.% resulted in fiber expansion during both charging and discharging, while the lower CDC contents (e.g., 25 wt.%) of the CNTCDC fiber were found to reduce expansion during the charging phase [21]. However, such mixed linear actuation is generally undesirable for practical applications, as it diminishes the overall actuation performance in terms of stress and strain. Ideally, a single, well-defined actuation direction is preferred for reliable operation. The primary advantages of CNT and CNTCDC fibers fabricated via the DEP method include their highly porous structure, which promotes enhanced charge density and improved capacitance. However, these fibers also exhibit brittleness, making them more suitable for integration into microelectromechanical systems. In this study, we investigated the effect of a lower CDC loading (10 wt.%) in CNT fibers, with a focus on their linear actuation behavior in both the polar aprotic (PC) and the polar protic (aqueous) solvents using the same electrolyte. Additionally, the energy storage performance of pristine CNT and CNTCDC fibers were compared in both solvent systems.

### 3.1. Characterizations of CNT and CNTCDC Fiber

The fibers were characterized using SEM, Raman spectroscopy, and the tensile stress–strain measurements under dry conditions and in the electrolytes with different solvents (PC and aqueous). The electrical conductivity of the CNT and CNTCDC fibers were also evaluated. EDX was performed on cross-sectional SEM images following approximately 120 linear actuation cycles per fiber, analyzed at both charged (+0.65 V) and discharged (−0.6 V) states to assess the elemental composition after electromechanical cycling.

**Figure 1:**
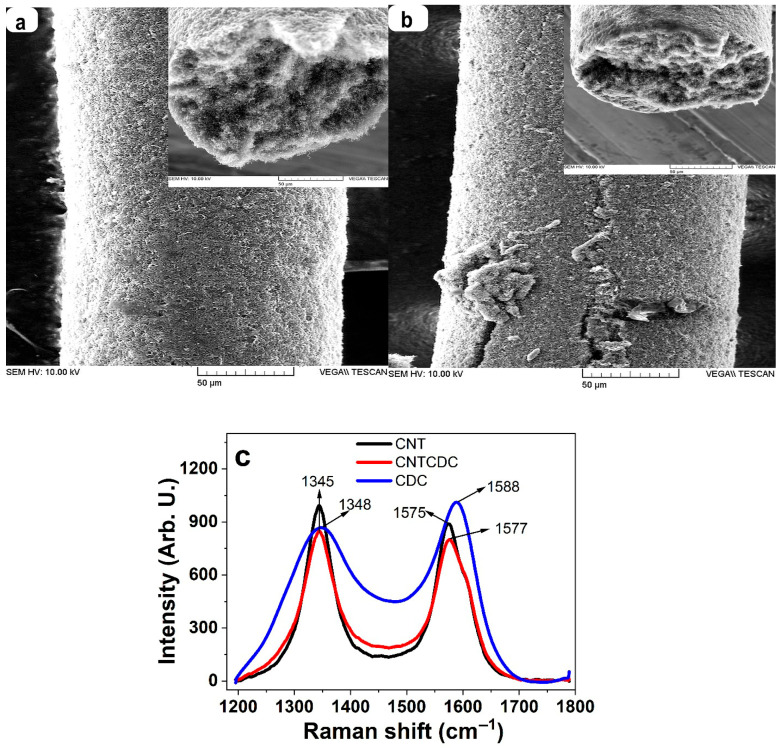
SEM images of the fiber (scale bar 50 µm) with an inset cross-section image of the CNT fiber are presented in (**a**), and the CNTCDC fiber is shown in (**b**). Raman spectroscopy (514 nm Argon ion laser) of the CNT fiber, the CDC particles, and the CNTCDC fiber are displayed in (**c**).

SEM images of the CNT and CNTCDC fibers, including their cross-sections, are presented in Figure 1a and Figure 1b, respectively. The corresponding Raman spectra are shown in Figure 1c. A higher-magnification SEM surface image of the CNT fiber is provided in Figure S1a, and the pore size distribution was analyzed using ImageJ software (Version 1.54g) [32], as shown in Figure S1b. The CNTCDC fiber surface is presented in Figure S1c, with the pore size distribution shown in Figure S1d, and the embedded CDC particle size analysis displayed in Figure S1e. High-resolution cross-sectional images of the CNT fiber are displayed in Figure S2. The CNTs within the CNT fiber exhibit an average length in the 1–2 µm range.

The SEM image of the CNT fiber (Figure 1a) showed a uniform surface, with the inset cross-sectional view indicating an average diameter of 149 µm. By comparison, the CNTCDC fiber (Figure 1b, inset) had a slightly larger diameter of 153 µm. The CNTCDC fiber surface appeared rougher due to the incorporation of CDC particles. Higher-resolution surface images (Figure S1a for CNT and Figure S1c for CNTCDC) revealed visible CNT chains and embedded CDC particles. Pore size evaluation using traditional BET analysis was not feasible due to the low mass of the individual fibers. Even with 33–35 fibers, the total mass reached only 6–7 mg, far below the 200 mg required for accurate BET measurements. As an alternative, surface pore size was estimated using ImageJ software [32] from the SEM images. The average pore size of the CNT fiber (Figure S1b) was 0.528 ± 0.1 µm, while the CNTCDC fiber showed a larger average pore size of 0.857 ± 0.13 µm (Figure S1d). The embedded CDC particles had an average diameter of 2.995 ± 0.207 µm (Figure S1e). The addition of CDC increased fiber porosity and raised the density of the CNTCDC fiber by approximately 3% compared to the pristine CNT fiber.

The enhanced porosity resulting from CDC incorporation also contributes to improved electrochemical properties. Previous studies [21] have shown that increasing the CDC content up to 50 wt.% led to even higher porosity and enhanced electronic surface conductivity. In the present study, electrical conductivity measurements in the dry state showed that the CNT fiber had a conductivity of 8.7 ± 0.6 S cm^−1^, while the CNTCDC fiber achieved 12.3 ± 1.1 S cm^−1^, about 1.4 times higher. It is well-known that greater alignment of CNTs improves conductivity, as demonstrated in ultrathin aligned SWCNT DEP-formed fibers (0.2–2 µm), which reached conductivities up to 200 S cm^−1^ [33]. By contrast, CDC-based materials typically require a binder to form stable structures. For example, cellulose-based CDC fibers (Cell-CDC) exhibit conductivities of just 0.21 ± 0.02 S cm^−1^ [34], and CDC films using PVdF-HFP as a binder only reach 0.4 ± 0.03 S cm^−1^ [35]. Thus, using CNTs as both the active and structural component for embedding CDC offers a significant advantage, yielding 30–60 times higher conductivity than binder-based composites.

The Raman spectroscopy results are shown in Figure 1c. The CNT fiber exhibited a narrow D-band at 1345 cm^−1^ (associated with the in-plane C–C bonding [36]) and a G-band at 1575 cm^−1^. The D-band appeared broader at 1348 cm^−1^ for the CDC particles, and the G-band was shifted to 1588 cm^−1^. In the CNTCDC fiber, the D-band remained nearly unchanged, while the G-band showed a slight upshift to 1577 cm^−1^. Previous research [37] has indicated that increasing the CDC content in CNT composites leads to a higher ID/IG ratio, indicative of increased structural disorder. Here, the CNT fiber had an ID/IG ratio of 1.025, while the CNTCDC fiber showed a slightly higher value of 1.07, reflecting minor defect formation due to non-uniform CDC particle size, as observed in Figure S1c,d.

**Figure 2:**
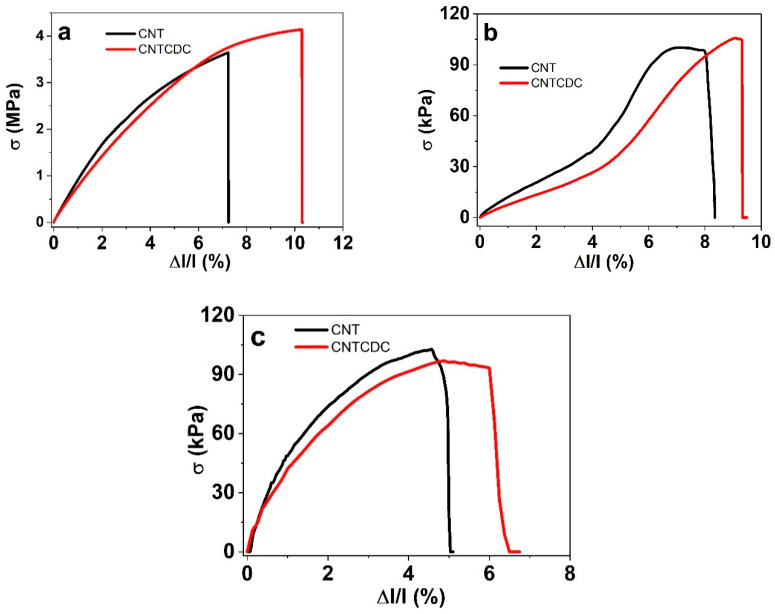
Stress σ against strain Δl/l (tensile strength) curves of CNT and CNTCDC fiber in the dry state shown in (**a**), in LiTFSI-PC in (**b**), and in LiTFSI-aq in (**c**).

Similar effects, such as increased porosity and structural irregularities, have been reported in other CDC/CNT composites [38], where large voids were observed. Nevertheless, the more open structure of the CNTCDC fiber is advantageous for ion transport, potentially enhancing charge–discharge performance compared to the pristine CNT fibers. Mechanical properties, including the tensile strength and the Young’s modulus, were evaluated using stress–strain curves under different conditions, including in the dry state (Figure 2a), in a polar aprotic solvent (PC, Figure 2b), and in a polar protic solvent (aqueous, Figure 2c).

The tensile stress against strain curves revealed that, in the dry state (Figure 2a), the CNT and CNTCDC fiber shapes were similar, as shown in previous research [21]. The DEP formation of the CNT fiber related to strength and thickness depends on the applied potential. When defects increase, they could cause a larger micro-void, decreasing the strength [14], due to impurities, such as CDC particle incorporation, disrupting the CNT alignment [39]. There was a general tendency, as shown in in Figure 2, that the CNT fibers had a higher modulus in the dry state.

The form of the stress–strain curve in Figure 2b show the different slopes at 3% strain, a much shallower one at 3.5% to 6% strain for the CNT fiber, with a larger slope. A similar tendency was found for the CNTCDC fiber in LiTFSI-PC. The fiber in LiTFSI-aq (Figure 2c) had a different profile, reflecting a minor increase in the CNT fiber modulus. Table 1 compares the tensile strength and the Young’s modulus of those fibers.

The influence of polar aprotic solvents, such as PC, have been investigated in previous research [20], which demonstrated that the inherently weak structure of the CNT fibers resulted in the low tensile strength and Young’s modulus. In the present study, the incorporation of 10 wt.% CDC into the CNT fibers improved both the tensile strength and elongation at break. However, the Young’s modulus in the dry state remained within a similar range—42.3 MPa for the CNTCDC fiber compared to 44.5 MPa for the CNT fiber (Table 1). Exposure to the solvents, whether PC or water (aq), reduced the tensile strength and the Young’s modulus for both fiber types. This decrease is primarily attributed to the behavior of CNTs within bundles [40], as shown in the earlier studies [21]. Increasing the CDC content up to 75 wt.% has been reported to cause a 50% reduction in the Young’s modulus, due to a weakening of the fiber’s internal structure.

At a lower CDC loading (10 wt.%), the presence of CDC particles introduces additional sliding between the CNTs and increases pore formation, disrupting the fiber matrix (Figure S1a) and reducing mechanical strength. Nonetheless, the tensile strength measurements in PC and the aqueous (aq) solvents (Table 1) showed no significant difference between the CNT and CNTCDC fibers. The Young’s modulus of the CNTCDC fibers was slightly lower than that of the CNT fibers in the LiTFSI-aq electrolyte, ranging from 0.9 to 1.03 MPa. By comparison, gelatin methacrylate hydrogels reinforced with aligned MWCNTs exhibited a considerably lower Young’s modulus, in the range of 21–23.4 kPa [41], which highlight the significantly higher stiffness of the CNT and CNTCDC fibers in this study.

**Figure 3:**
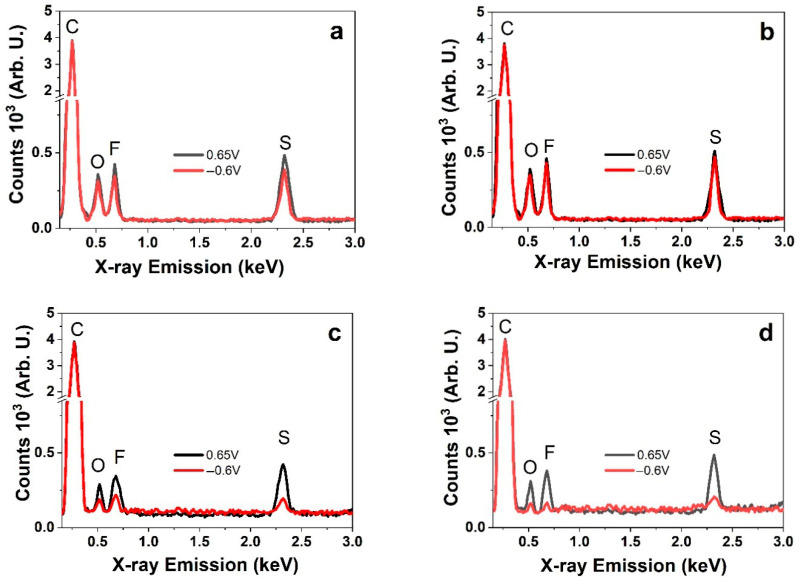
EDX spectra of cross-section image at charged (0.65 V) and discharged (−0.6 V) after linear actuation, showing in (**a**) for CNT and in (**b**) for CNTCDC fiber in LiTFSI-PC. The EDX spectra after actuation in LiTFSI-aq are presented in (**c**) for CNT and (**d**) for CNTCDC fiber.

Further analysis to determine the element composition after linear actuation at charged condition (0.65 V) and discharged condition (−0.6 V) were performed by EDX spectroscopy (cross-section SEM images). The CNT and CNTCDC fiber after actuation in LiTFSI-PC are shown in Figure 3a,b, and those in LiTFSI-aq actuated samples regarding EDX analysis are presented in Figure 3c,d.

The dominant carbon signal (C) at 0.26 keV, the oxygen signal (O) at 0.52 keV, the fluoride (F) signal at 0.68 keV, and the sulfur signal (S) at 2.32 keV were shown in all EDX spectra in Figure 3a–d. The element variation at charging (0.65 V) and discharging (−0.6 V) for the CNT fiber in the LiTFSI-PC electrolyte (Figure 3a) showed a decrease in intensity for sulfur, fluoride, and oxygen signals. Due to its small size, the Li element was not detectable in this EDX spectrum. Hence, the elemental fluoride, oxygen, and sulfur signals mainly belonged to the electrolyte LiTFSI, and changes in intensity hinted at the incorporation of TFSI^−^ anions at charging, while some are removed at discharging. The CNTCDC fiber in LiTFSI-PC in Figure 3b revealed only a minor sulfur, fluoride, and oxygen change during discharging. Previous research [21] has shown that 25 wt.% in CNTCDC fiber for CDC content was similar to the EDX spectra shown in Figure 3b. It was concluded from this research that signals of sulfur, fluoride, and oxygen in minor changes at charging refer to some TFSI^−^ incorporation that did not move out during discharging.

Consequently, EDL was formed during discharging through the association with Li⁺ ions. In this study, as illustrated in Figure 3a, the observed fluctuations in elemental composition during charging and discharging suggested the involvement of multiple ion species. Elemental analysis of CNT and CNTCDC fibers in the LiTFSI-aqueous (aq) electrolyte (Figure 3c,d) revealed distinct behavior. For the CNT fiber (Figure 3c), a pronounced increase in the signal intensity of oxygen, fluorine, and sulfur was detected during charging at +0.65 V. Upon discharging, these signals diminished significantly, indicating that these elements, associated with TFSI^−^ anions, were primarily involved in EDL formation during charging. A small fraction of TFSI^−^ anions remained within the CNT fiber after discharge.

A similar trend was observed for the CNTCDC fiber (Figure 3d), although trace amounts of sulfur, fluorine, and oxygen were still detectable during discharging. Comparable behavior had been reported for cellulose, MWCNT composites fabricated via extrusion and tested in aqueous systems, even when different electrolytes were used [42]. It suggests the following general tendency in CNT-based fibers: the elemental changes during the charge/discharge cycles are influenced by the solvent type, polar aprotic or polar protic, which governs whether electrolyte anions remain embedded or are expelled from the fiber structure [27].

### 3.2. EMD Measurements of CNT and CNTCDC Fiber

The CNT and CNTCDC fibers, when operated as capacitors within the applied potential window of +0.65 V to −0.6 V, function as actuators through a non-faradaic mechanism [23], where charge injection was balanced by the formation of an EDL, resulting in ion flux. This study focused on the effect of solvent polarity, using the same salt (LiTFSI) in both a polar aprotic solvent (PC) and a polar protic solvent (water, aq), on DEP-formed CNT and CNTCDC fibers. Various electrochemical techniques were employed to investigate their linear EMD behavior. In addition, the energy storage capabilities of these materials were evaluated through chronopotentiometric measurements.

#### 3.2.1. Cyclic Voltammetric Studies

**Figure 4:**
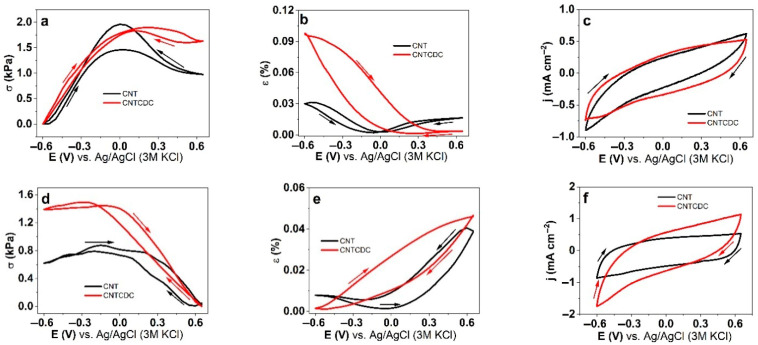
Cyclic voltammetric (scan rate 5 mV s^−1^) EMD measurements of CNT fiber (black line) and CNTCDC fiber (red line) showing in (**a**) stress σ, (**b**) strain ε, and (**c**) current density j against potential E (0.65 V to −0.6 V) in LiTFSI-PC electrolyte. Applying LiTFSI-aq electrolyte on CNT and CNTCDC fiber with stress σ in (**d**), strain ε in (**e**), and current density j in (**f**). The arrows in the figures are showing the direction of the scan of the third cycle. The lowest stress value was set to zero, and the strain was always opposite to the stress. The expansion in stress refers to the contraction in strain.

Cyclic voltammetry (scan rate 5 mV s^−1^) of the CNT and CNTCDC fibers in the polar aprotic solvent PC and the polar protic solvent water (aq) using the same salt LiTFSI at the potential range 0.65 V to −0.6 V are shown in Figure 4. The stress and strain potential curves of the CNT and CNTCDC fibers in LiTFSI-PC are presented in Figure 4a and Figure 4b, respectively. The current density curves are shown in Figure 4c, and the charge density curves (coulovoltammetry) of the CNT fiber are displayed in Figure S3a. The CNT and CNTCDC fiber in the LiTFSI-aq electrolyte regarding stress is shown in Figure 4d, strain in Figure 4e, and current density in Figure 4f. The coulovoltametric response is presented in Figure S3b.

A comparison of the stress–strain curves of the CNT and CNTCDC fibers in LiTFSI-PC (Figure 4a,b) revealed that the CNT fibers exhibit mixed actuation, with the primary expansion occurring during discharge (Figure 4b). By contrast, the CNTCDC fibers showed a dominant expansion during discharge. However, when tested in the LiTFSI–aqueous (aq) electrolyte, the actuation direction reversed, with the CNT and CNTCDC fibers showing primary expansion during charging. A minor expansion was also observed during discharge for the CNT fibers. EDX spectroscopy of the CNT fibers in LiTFSI–PC indicated elemental changes (fluoride, oxygen, and sulfur) during charging and discharging, suggesting mixed ion movements. Previous studies [20] have proposed that larger anions, such as TFSI^−^ or triflate (CF_3_SO_3_^−^), can be incorporated into the smaller pores (less than 1 nm) of CDC within the CNTCDC fibers more readily than in the CNT fibers, which have larger pore sizes (3–6 nm, as reported by the supplier) [43]. This incorporation favored expansion during discharge, when Li^+^ ions (along with solvent molecules) form the EDL.

Earlier research [44] has pointed out that CNT in fiber or solid electrodes followed a faradaic process, merely over observed redox peaks in cyclic voltammetry. However, another study [45] attributed minor charging/discharging signals in CNTs to impurities, mainly referred to as iron particles that came from the catalyst of the chemical vapor deposition (CVD) of CNT. The CNT fiber and the CNTCDC fiber (Figure 4c,d) in our research had capacitive current density shapes, while the CNTCDC fiber had higher current density due to higher electronic conductivity (1.3 times higher). The charging/discharging curves of the CNT fiber (Figure S3a) and the CNTCDC fiber (Figure S3b) showed that charging/discharging is in balance over the closed loops. Those steady-state conditions are generally applied for faradaic actuators, such as conducting polymers, where over-oxidation or over-reduction could lead to uncontrollable actuator response [46]. In the case of the CNT and CNTCDC fibers, avoiding irreversible reactions was the main reason for keeping charging/discharging in balance. Table 2 compares the stress, strain, and charge density of the CNT and CNTCDC fiber in the LiTFSI salt using the solvents PC and aq.

The CNT fiber (Table 2) showed, either in LiTFSI-PC or in LiTFSI-aq, mixed linear actuation, while the CNTCDC fiber had main expansion at discharging in LiTFSI-PC and main expansion at charging in LiTFSI-aq. Previous research [2] using MWCNT twist spun yarn in organic electrolyte (polar aprotic solvent, acetonitrile (ACN)) also revealed mixed actuation with main expansion at discharging and minor expansion at charging, showing strain up to 0.2%. Other research [47], which used the same polar aprotic solvent and applied MWCNT paper, showed a strain in the same range with expansion at discharging. Organic electrolytes using the polar aprotic solvents (ACN) of the direct spun CNT yarns showed mixed linear actuation [18] with expansion at discharging (−1.0 V), with a strain of 0.035% and similar values for strain at charging. The explanation was given partly due to anion incorporation in the CNT pores in the yarn, leading to cation and EDL formation at discharging.

Using a polar protic solvent (aq), applying CNT mat (MWCNT) showed a linear strain of 0.15–0.2% with main expansion at discharging in the aqueous electrolyte [48]. If there was strain at discharging, the EDL is formed of entrapped anions, with explanation seeing such in the aqueous solvent referred to as denser SWCNT or MWCNT mats with only outer surfaces in contact, while the inner cores are not involved [48]. Recent research [49] has confirmed that CNT buckypaper actuation properties in the aqueous electrolyte depend on the applied ion size. Our CNT fibers are very porous, with expected enhanced ion and solvent penetration better than other CNT yarns or mats. In the case of CNTCDC, the porosity, as shown in Figure S1d, was enhanced compared to the CNT fiber (Figure S1b).

The stress of the CNTCDC fiber was 1.7 times higher, and the strain difference was 6.7 times higher in LiTFSI-PC. In LiTFSI-aq at charging, the stress was 2.2 times higher in the CNTCDC fiber and 1.5 times higher than for the CNT fiber. The charge density for the CNTCDC and CNT fibers in LiTFSI-PC was 1.7 times higher, and those in LiTFSI-aq had 1.5 times higher values. With a higher amount of CDC in the CNT fiber, as shown from previous research [21], the stress and strain increased to 50 wt% CDC content in the CNT fiber, mainly expanding at discharging in LiTFSI-PC but also had minor expansion at charging. Overall, a lower CDC amount, such as 25 wt.%, showed that expansion at charging was reduced, which also presented in this work that 10 wt.% CDC in the CNT fiber seemed to be the choice for the main expansion during discharging. Previous research using CDC with PVdF-HFP sandwiched between fiberglass forming a trilayer investigated as linear actuators in LiTFSI-PC had the main expansion during discharging at a range of 0.3% strain. The main explanation was given for the entrapped anions in the CDC pores, as observed in other research [26], where asymmetric swelling of CDC electrodes in ionic liquids occurred.

Figure 4 and Table 2 reveal that mixed linear actuation is not preferable for any linear actuator with an overall limited strain and stress outcome. Further analysis applying EMD chronoamperometry measurements were conducted.

#### 3.2.2. Chronoamperometric EMD Measurements

**Figure 5:**
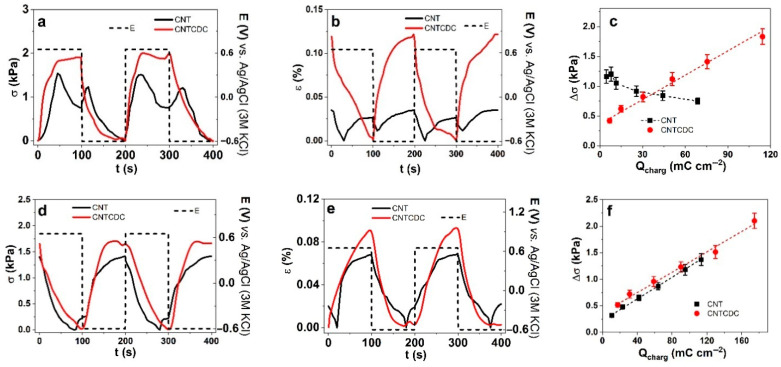
EMD square potential step measurements of CNT (black line, ■) and CNTCDC fiber (red line, ●) in LiTFSI-PC showing two subsequent cycles (3rd to 4th 0.005 Hz) of stress σ in (**a**) and strain ε in (**b**) at the potential range E (dashed line, 0.65 V to −0.6 V). The stress difference Δσ against charge density (Q_charg_) is presented in (**c**). The similar follow-up of CNT and CNTCDC fibers in LiTFSI-aq of stress–time curves are shown in (**d**), strain time curves are displayed in (**e**), and stress difference Δσ against charge density is shown in (**f**). The dashed lines in (**c**,**f**) represent the linear fit.

The CNT and CNTCDC fibers underwent chronoamperometric EMD measurements at applied frequencies ranging from 0.0025 Hz to 0.1 Hz within a potential window of 0.65 V to −0.6 V. The stress–time curves recorded at 0.005 Hz for the CNT and CNTCDC fibers in LiTFSI-PC (a polar aprotic solvent) are shown in Figure 5a, while the corresponding strain–time curves are presented in Figure 5b. From the current density–time curves (Figure S4a), the charge density was calculated by integration at each applied frequency. The resulting relationship between the stress difference and the charge density is shown in Figure 5c. In the LiTFSI-aqueous (aq, a polar protic solvent) electrolyte, the stress profiles of two consecutive cycles for both fiber types are displayed in Figure 5d, and the corresponding strain profiles are shown in Figure 5e. Similarly, charge densities at each frequency are calculated from the current density–time curves (Figure S4b), and the relationship between stress difference and charge density is plotted in Figure 5f. The dependence of stress and strain differences on applied frequency for the CNT and CNTCDC fibers in LiTFSI-PC are shown in Figure S4c,d, respectively. For the LiTFSI-aq system, the corresponding data are presented in Figure S4e,f.

The stress–time curves of the CNT fiber (Figure 5a) in LiTFSI-PC (PC, polar aprotic solvent) had mixed linear actuation with stress at charging (0.65 V) increasing at 47 s to 1.55 kPa and then decreasing at 100 s to 0.75 kPa. During the discharging process, the stress decreased further to 0 kPa. The lowest point in the stress–time curves was set to zero, and a decrease in stress referred to an increase in strain. The strain was always the opposite of stress in the linear actuation measurements [29], as observed in Figure 5b. Mixed linear actuation, such as CNT fiber, had lower stress and strain differences, as shown in Figure 5c and Figure S5a. In the case of the CNTCDC fiber, the main expansion during discharging was observed. It also needs to be noted that, at different potential ranges (1.0 V to −0.8 V), as chosen in former research [21], mixed linear actuation took place for CNTCDC (with increasing CDC loads). With 10% of the CDC applied in the CNT fiber, no significant expansion in charging was observed in this work. The stress difference against charge density shown in Figure 5c revealed some odd behavior for the CNT fiber with a similar tendency of stress difference against frequency in Figure S4c and strain difference against charge density in Figure S5a.

At a low charge density, the stress difference had its maximum with a further decrease at a higher charge density (lower frequency). The reason for such behavior has been reflected in recent research [28], which shifted from mixed actuation at a high charge density to main expansion at discharging. The TFSI^−^ anions are incorporated in the CNT pores and stayed partly inside at a high charge density (low frequency). The longer time led to some TFSI^−^ anions leaving the CNT pores during discharging. That is why we observed an extension in discharging and charging. At a low charge density (max stress at 0.05 Hz, Figure S5a), the discharging time was reduced, while most TFSI^−^ stayed inside the CNT pores. To balance the charge of those trapped anions during discharging, Li^+^ ions with the solvent formed the EDL, with then led to expansion at discharging.

In the case of the CNTCDC fiber (Figure 5a,b), CDC’s reduced pore size guided to more trapped TFSI^−^ anions, with main expansion at discharging over EDL formation with Li^+^ cation. This was observed in Figure 5c and Figure S5a, with nearly linear stress and strain differences against charge densities. Riemenschneider et al. [50] studied the theoretical and experimental effect of CNT buckypaper having high capacitance, leading in general electrolytes to high strain, with that strain reduced at lower time. Such charge dependency on linear stress or strain behavior has been shown in prior studies using CNTCDC (30%) fiber in different electrolytes with the same solvent [20].

In LiTFSI-aq (polar protic solvent), the linear actuation direction of the CNT and CNTCDC fiber changed to main expansion at charging (Figure 5d,e), which was also observed in cellulose MWCNT fiber [27]. The main explanation of expansion at discharging in LiTFSI-PC (Figure 5a,b) and expansion at charging in LiTFSI-aq (Figure 5d,e) were given due to the lower dipole moment of the aqueous solvent (water has 1.85 D and PC has 4.94 D) and the nearly 93% wettability [51] of the fiber, leading at charging to the TFSI^−^ EDL formation. Most TFSI^−^ (fluoride elements at EDX spectra, Figure 3d) left the CNTCDC fiber during discharging. In the case of the CNT fiber, the stress curves in Figure 5d and the strain curves in Figure 5e show mixed linear actuation. Also comparable to the CNT fiber in LiTFSI-PC, the mixed actuation showed small expansion at discharging, with the main expansion at charging. The stress difference (Figure 5f) and strain difference (Figure S5b) revealed the linear dependency of the CNT and CNTCDC fibers against the charge density. The stress difference (Figure S4e) and the strain difference (Figure S4f) showed that the CNTCDC fiber had higher stress and strain than the CNT fiber. The stress difference and the strain difference at frequencies 0.0025 Hz and 0.05 Hz (Figure S4c) correlated to the charge densities of the CNT and CNTCDC fibers (Figure 5c,f and Figure S4a,b) using the same salt LiTFSI in the polar aprotic solvent (PC) and the polar protic solvent (aq) are compared in Table 3.

The charge density at charging (Table 3) of the CNTCDC fiber in LiTFSI-PC and LiTFSI-aq was 1.5–1.6 times larger than that of the CNT fiber. One reason for such a higher charge density was the 1.4 times higher electronic conductivity of the CNTCDC fiber. The larger surface area, the smaller pore size of CDC, and the higher density of the CNTCDC fiber affect electron transport, increasing the charge density, as has been shown before [21]. Despite the CNT fiber in LiTFSI-PC, where the stress and strain increased with a lower charge density (Table 3), the CNTCDC fiber showed a more predictable stress and strain linearity against the charge density (Figure 5c and Figure S5a). The overall stress difference of CNTCDC in LiTFSI-aq at 2.5 mHz and 50 mHz was 1.5 times enlarged, and the strain difference reached 1.7 to 1.9 times higher values. Therefore, the CNTCDC fiber in both solvents, polar aprotic (PC) and polar protic (aq), using the same salt (LiTFSI), was more controllable in linear actuation with higher stress, strain, and charge density. The DEP process forming CNT fiber, as shown in previous research [52], can control the density of the fiber and its alignment. Thinner (1 µm diameter) and denser SWCNT fibers have been proposed for high electrochemical capacitors [33]. The CNT and CNTCDC fibers are subject to energy storage-related materials, which are investigated in the next section.

### 3.3. Energy Storage of CNT and CNTCDC Fiber

The ability of MWCNTs (CNTs) to store energy [53] is of great interest due to their stability, sustainability, flexibility, and high conductivity. CNT as wet-spun fibers [1] showed specific capacitance in the aqueous electrolytes in the range of 70 F g^−1^ (0.5 A g^−1^) while flexible buckypaper [54] sheets can reach a specific capacitance of 270 F g^−1^ at the scan rate of 2 mV s^−1^. CNTs are electrical double-layer capacitors (EDLC) with the basic mechanism of storing charge obtained electrostatically from ion adsorption [55]. The surface area, electronic conductivity, packed density, and pore size of the CNT electrodes are essential to reach a high charge density storage with good access to ion mobility in the provided electrolyte [56]. DEP-formed CNT fibers are well-known for their increased surface area, porous nature, and moderate electronic conductivity. CNT-based composites for energy storage are mainly investigated in the aqueous electrolytes (polar protic solvent) due to the better dielectric constant of water. It can facilitate ion solvation and transport, affecting the charge storage capability. CNT also had nearly 93% wettability in water [51], facilitated better anion water transport [57], while cation water transport was insignificant. From linear actuation studies (Figure 5), the charge density in aqueous electrolytes was found to be 1.6 times higher in comparison to the polar aprotic solvents and nearly 1.5 times higher for the CNTCDC fiber.

**Figure 6:**
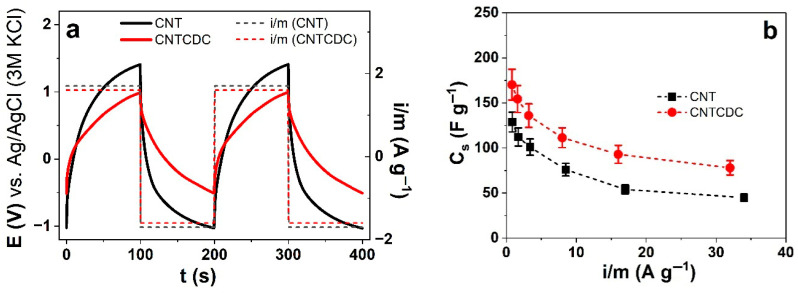

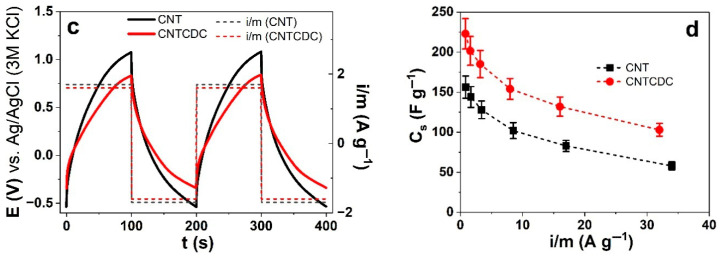
Chronopotentiometric measurements of CNT (black line, --■--) and CNTCDC fiber (red line, --●--) with potential time curves (±1.6 A g^−1^, dashed black and ±1.7 A g^−1^ dashed red line) of two subsequent cycles (3rd to 4th) using same salt with showing in (**a**) the polar aprotic (PC) solvent. The specific capacitance C_s_ against varied current densities i/m for CNT and CNTCDC fiber are presented in (**b**). In the polar protic solvent (aq), the potential time curves are presented in (**c**), and the specific capacitance against current densities i/m is shown in (**d**).

Taking the charge density constant at varied current densities, chronopotentiometric measurements of the CNT and CNTCDC fibers were performed. The potential time curves for the CNT fiber in the polar aprotic solvent (LiTFSI-PC) at ±1.6 A g^−1^ (charge density ± 160 C g^−1^) and the CNTCDC fiber at ±1.7 A g^−1^ (±170 C g^−1^) are presented in Figure 6a. At each applied current density, the obtained potential time curves of the slope at discharging (after IR drop) are taken to calculate the specific capacitance (Equation (1)), C_s_. The results for the LiTFSI-PC electrolyte are presented in Figure 6b. When the polar protic solvent (LiTFSI-aq) is applied to the CNT and CNTCDC fibers, the potential time curves are shown in Figure 6c, and the specific capacitance against the applied current densities is displayed in Figure 6d. To evaluate the specific capacitance retention of the CNT fiber (34 A g^−1^) and the CNTCDC fiber (32 A g^−1^), 5000 cycles are performed with the results for the polar aprotic solvent (PC) shown in Figure S6a and the polar protic solvent (aq) in Figure S6b. From each CNT and CNTCDC fiber, at least three different fibers are formed and measured, with the results shown in mean values with standard deviation.

In general, as seen in Figure 6a (polar aprotic solvent, PC), high potential evolution for the CNT fiber (1.41 V) was reflected in higher resistivity (lower conductivity) in comparison to the CNTCDC fiber (0.99 V). The two subsequent cycles, if overlapping for each fiber, CNT or CNTCDC, are concurrent, showing that charging/discharging was in balance [28]. More important is the slope of the potential time curves at discharging, which is shallower for the CNTCDC fiber than the CNT fiber. The specific capacitance (obtained from Equation (1)) against the applied current density, as shown in Figure 6b, revealed that, with the increasing current density, the specific capacitance decreased. The best specific capacitance of 170.3 ± 13 F g^−1^ is found for the CNTCDC fiber at ± 0.8 A g^−1^ (Figure 6b), while the CNT fiber at 0.85 A g^−1^ has 128.8 ± 11 F g^−1^.

Previous research [21] using maximum loads of CDC (75 wt.%) in the CNT fiber showed 175.5 ± 17 F g^−1^ (± 0.54 A g^−1^). Pure CDC composite using (PVdF-HFP) as polymeric binders was hot pressed together with embedded ionic liquids [58], revealed specific capacitance of 119 F g^−1^ (1 mV s^−1^). The combination of CNT with CDC using a sol–gel process of TiC-CDC with 1% and 2% CNT revealed the best specific capacitance of 112 F g^−1^ (10 mV s^−1^) [59] in the organic electrolyte using acetonitrile (ACN) as a solvent, while the addition of CNT up to 2% reduced the capacitance to 93 F g^−1^. Directly electrospun CDC fiber in ionic liquids and ACN solvent showed specific capacitance at 105 F g^−1^ [60]. Directly drawn MWCNT yarn [2] had a specific capacitance of 26 F g^−1^ (1000 mV s^−1^) in the organic electrolyte using the polar aprotic solvent (ACN). The combination of CNT with CDC made over the DEP process revealed much better specific capacitance than wet-spun fiber or directly drawn yarns.

The specific capacitance for the CNT fiber (Figure S6a) at 34 A g^−1^ (5000 cycles) at cycle 5 showed 45 ± 4 F g^−1^ and 38.5 ± 3.4 F g^−1^ at cycle 5000, which gave a capacity retention of 85.3%. MWCNT coated on cellulose fibers (CF), which are then carbonized (CCF), revealed in the organic electrolytes a specific capacitance of 156 F g^−1^ (50 mA g^−1^) with 2000 cycle stability of 84% (400 mA g^−1^) [61], similar to our CNT fiber results. High specific capacitance retention was found for CNT coated on carbon fiber cloth in the organic electrolytes with solvent ACN, which showed 1.56 F g^−1^ up to 100.000 cycles with no decrease of capacitance [62].

For the CNTCDC fiber (Figure S6a), the specific capacitance at 32 A g^−1^ is 78 ± 6.3 F g^−1^, with that at cycle 5000 showing 71.6 ± 6.4 F g^−1^, having a specific capacitance retention of 91.7%. Previous research [21] using CNTCDC with 50 wt.% CDC showed specific capacitance retention at ± 27.9 A g^−1^ of 85% after 1000 cycles. Mixtures with other carbon materials and CNT, such as MXene/MWCNT [63] in the organic electrolytes, revealed 90% capacity retention after 1000 cycles. The combination of activated carbon (AC) with CNT in the organic electrolytes [64] had a specific capacitance of 170 F g^−1^ with a capacity retention of 93% after 100,000 cycles.

The change from the polar aprotic (PC) to the polar protic (aq) solvent affects the CNT energy storage, which is observed in higher specific capacitance. In the polar protic solvent (aq), the potential time curves (Figure 6c) showed a similar tendency of higher potential evolution of 1.1 V at ± 1.7 A g^−1^ for the CNT fiber, while the CNTCDC fiber had 0.88 V at 1.6 A g^−1^. The overall slope from the CNT fiber potential time curve at discharging was 1.4 times larger than that from the CNTCDC fiber. The best specific capacitance (Figure 6d) is presented for the CNTCDC fiber with 223 ± 17 F g^−1^ (± 0.8 A g^−1^) with capacity retention (Figure S6b) at 32 A g^−1^ of 94.2 % (cycle 5: 103 ± 9.4 F g^−1^, cycle 5000: 97 ± 8.9 F g^−1^). The CNT fiber had a much lower capacitance of 156.4 ± 14 F g^−1^ (± 0.85 A g^−1^), as shown in Figure 6d with a capacity retention of 82.7% (Figure S6b) at 34 A g^−1^ (cycle 5: 58 ± 5.1 F g^−1^, cycle 5000: 48 ± 4.4 F g^−1^).

CNT in the aqueous electrolytes could reach a specific capacitance between 5 and 135 F g^−1^ [65]. Numerous research studies [66] have been conducted using carbon-based fiber with CNT yarn. MWCNT yarn, such as yarn drawn from forests, is mainly used in a twisted form, having high conductivity above 300 S cm^−1^ [17] with specific capacitance in aqueous gels (polyvinyl alcohol, PVA, with H_3_PO_4_) of 86.2U F g^−1^ [10].

In most cases, EDL-based MWCNTs are combined with pseudocapacitors, such as conducting polymers or metal oxides, aiming to have a higher stored charge density and superior capacitance [4]. A combination of MWCNT yarn with polypyrrole (PPy) from prior research [67] revealed the low specific capacitance of pristine MWCNT of 40 F g^−1^, with chemically oxidized PPy having 59 F g^−1^ and electrochemically deposited at 53 F g^−1^ in the aqueous electrolytes. Much higher specific capacitance in the electrochemical deposition of MnO_2_ on MWCNT wet spun fiber in the aqueous electrolytes showed the specific capacity of 152 F g^−1^ and 16% cyclic retention after 10,000 cycles [68].

As a summary, Table S1 compares the fiber or yarn CNT and CDC composites in their actuation properties and their capacitance. There are only a few examples for the MWCNT fiber found in dual functionality of actuator and supercapacitor. In general, high strain either for yarn or fiber led to low capacitance, as shown for the twist spun MWCNT yarn with best strain of 0.5% at −2.5 V in the organic electrolytes, while the capacitance was in the range of 26 F g^−1^ [2]. Similar results in capacitance were found for graphene CNT yarn [69] (22.6 F g^−1^) with strain at discharging of 0.05%. Electro spun CDC fiber [60], MWCNT yarn with MnO_2_ [70]_,_ or carbon yarn filled with activated carbon [71] have been applied as supercapacitor materials but no actuation has been reported. The combination of DEP-formed CNT and CNTCDC fiber in the polar aprotic and the polar protic solvents in this study showed dual functionality as an actuator and supercapacitor. The CNTCDC fiber having high specific capacitance with high capacity retention, making such fiber applicable in flexible energy storage devices.

## 4. Conclusions

DEP formed a thick CNT fiber with a diameter of 149 µm and with 10 wt.% CDC the CNTCDC fiber in a diameter of 153 µm was formed. The SEM images revealed porous CNT and CNTCDC fiber with a Young’s modulus in the dry state were similar at the 42–44 MPa range. Then, in the polar aprotic (PC) and the polar protic (aq) solvents, the Young’s modulus was reduced to an average of 0.8–1.05 MPa. Their linear EMD measurements applying cyclic voltammetry following the EDL process revealed the CNT fiber in the polar aprotic solvents (PC) using the LiTFSI salt, mixed actuation with main expansion at discharging (strain 0.03%) due to entrapped TFSI^−^ anions in the CNT pores (confirmed over EDX element detection of sulfur, fluoride, and oxygen at discharging). The CNTCDC fiber had only minor expansion at charging and main expansion at discharging with a strain of 0.1%. The actuation direction changed in the polar protic solvent (aq, with LiTFSI salt) of the CNT fiber to main expansion at charging (strain of 0.04%) with still minor expansion at discharging, while the CNTCDC fiber showed 0.047% strain with main expansion at charging. The overall charge density of CNTCDC compared to CNT in both solvents is 1.5–1.6 times enhanced, which is reflected in the 1.4 times better conductivity. Applying chronopotentiometric measurements, the specific capacitance revealed the best values in the polar protic solvent for the CNTCDC fiber of 223 ± 17 F g^−1^ (± 0.8 A g^−1^) with capacity retention after 5000 cycles at 32 A g^−1^ of 94.2%. In the polar aprotic solvent, the CNTCDC fiber has 170.3 ± 13 F g^−1^ with capacity retention at 91.7%. The specific capacitance of the CNT fiber in the polar aprotic and the polar protic solvents is found to be 1.3–1.4 times reduced, with an overall capacity retention of 85–82% at 34 A g^−1^. The CNTCDC fiber is superior to the CNT fiber in actuators and energy storage devices. Further research using different electrolytes is envisaged to elaborate on potential sensor functionality.

## Figures and Tables

**Table 1:** CNT and CNTCDC fiber elongation at break A, tensile strength σ_T_, and Young’s modulus Y in dry state and in both the polar aprotic (PC) and the polar protic (aq) solvents using the same salt LiTFSI.

Parameters	A (%)		σ_T_ (MPa)		Y (MPa)	
	CNT	CNTCDC	CNT	CNTCDC	CNT	CNTCDC
dry	7.2 ± 0.4	10.3 ± 0.6	3.6 ± 0.2	4.1 ± 0.2	44.5 ± 2.6	42.3 ± 0.3
LiTFSI-PC	8.0 ± 0.5	9.1 ± 0.5	0.1 ± 0.01	0.11 ± 0.01	0.88 ± 0.07	0.72 ± 0.04
LiTFSI-aq	4.6 ± 0.2	6.0 ± 0.3	0.1 ± 0.01	0.1 ± 0.01	1.03 ± 0.1	0.9 ± 0.06

**Table 2:** Strain ε actuation parameters at charging (0.65 V) and discharging (−0.6 V) and stress differences (Δσ), as well as the charge density Q of CNT and CNTCDC fibers in the electrolyte LiTFSI with the solvents PC and aq.

Fiber	Δσ (kPa)	ε (%)		Q (mC cm^−2^)
		0.65 V	−0.6 V	
CNT (PC)	0.97	0.017	0.031	45.6
CNTCDC (PC)	1.64	0.004	0.1	77.4
CNT (aq)	0.62	0.04	0.008	86.7
CNTCDC (aq)	1.39	0.047	0	131

**Table 3:** Stress difference (Δσ), strain difference Δε, and charge densities at charging Q_charg_ of the CNT and CNTCDC fibers in LiTFSI-PC and LiTFSI-aq at 2.5 mHz and 50 mHz frequency. The parameters shown in the Table are mean values with a standard deviation between 8 and 12%.

Fiber	LiTFSI-PC			LiTFSI-aq		
	Δσ (kPa)	Δε (%)	Q_charg_ (mC cm^−2^)	Δσ (kPa)	Δε (%)	Q_charg_ (mC cm^−2^)
CNT						
2.5 mHz	0.75	0.007	70.8	1.37	0.056	113.4
50 mHz	1.21	0.019	7.8	0.48	0.021	23.2
CNTCDC						
2.5 mHz	1.83	0.152	114.4	2.13	0.095	174.7
50 mHz	0.62	0.348	14.8	0.72	0.04	31.2

## Data Availability

The original contributions presented in this study are included in the article/Supplementary Materials. Further inquiries can be directed to the corresponding author.

## Supplementary Materials

The following supporting information can be downloaded at: https://www.mdpi.com/article/10.3390/ma18143254/s1, Figure S1: Analyse using ImageJ software on SEM images (scale bar 10 µm) showing the surface of CNT fiber in (a). The average pore size analysis (diameter of pores, d) of CNT fiber is presented in (b). The SEM surface image of CNTCDC is displayed in (c) and the circles showing the CDC particles, with the diameter of pores shown in (d). The CDC’s particle size (showing diameter d) in CNT is presented in (e); Figure S2: SEM image (scale bar 5 µm) of CNT fiber higher cross-section resolution with average CNT length at the range of 1–2 µm; Figure S3: Coulovoltametry of 3rd cycle (charge density Q against potential E) of CNT fiber (black line) and CNTCDC fiber (red line) measurements in LiTFSI-PC presented in (a) and LiTFSI-aq in (b). The arrows show the direction of the scans; Figure S4: EMD chronoamperometric measurements of CNT and CNTCDC fiber of current density time curves against time (cycle 3rd to 4th) are presented for LiTFSI-PC in (a) and LiTFSI-aq in (b). The stress and strain difference in LiTFSI-PC of CNT and CNTCDC fiber against the applied frequencies f are shown in (c) and (d). The stress and strain differences in LiTFSI-aq against frequencies are presented in (e) and (f); Figure S5: Square wave steps combined with EMD measurements of CNT (black line, ■) and CNTCDC fiber (red line, ●) at potential range E (0.65V to -0.6V, dashed line) showing in (a) strain differences Δε against charge density Q_charg_ in LiTFSI-PC and in (b) strain differences Δε in LiTFSI-aq against charge density in (b). The dashed lines represent the linear fit, showing for orientation only; Figure S6: Long-term chronopotentiometry measurements showing specific capacitance Cs against cycles (5000 cycles) of CNT (■) at 32 A g^−1^ and CNTCDC (●) fiber at 34 A g^−1^ with those shown in polar aprotic solvent (PC) in (a) and polar protic (Aq) in (b); Table S1: Comparison of carbon nanotubes (CNT) and carbide-derived carbon (CDC) composites in fiber actuation and their specific capacitance C_s_, capacitance retention (CR) in organic and aqueous electrolytes. References [2,60,69,70,71,72] are cited in the Supplementary Materials.

## References

[1] Lu Z., Chao Y., Ge Y., Foroughi J., Zhao Y., Wang C., Long H., Wallace G.G. (2017). High-Performance Hybrid Carbon Nanotube Fibers for Wearable Energy Storage. Nanoscale.

[2] Mirfakhrai T., Oh J., Kozlov M., Fok E.C.W., Zhang M., Fang S., Baughman R.H., Madden J.D.W. (2007). Electrochemical Actuation of Carbon Nanotube Yarns. Smart Mater. Struct..

[3] Qi H., Schulz B., Vad T., Liu J., Mäder E., Seide G., Gries T. (2015). Novel Carbon Nanotube/Cellulose Composite Fibers As Multifunctional Materials. ACS Appl. Mater. Interfaces.

[4] Ghosh A., Lee Y.H. (2012). Carbon-Based Electrochemical Capacitors. ChemSusChem.

[5] Zhu Y., Yue H., Aslam M.J., Bai Y., Zhu Z., Wei F. (2022). Controllable Preparation and Strengthening Strategies towards High-Strength Carbon Nanotube Fibers. Nanomaterials.

[6] Zhang X., Lu W., Zhou G., Li Q. (2020). Understanding the Mechanical and Conductive Properties of Carbon Nanotube Fibers for Smart Electronics. Adv. Mater..

[7] Lu W., Zu M., Byun J.H., Kim B.S., Chou T.W. (2012). State of the Art of Carbon Nanotube Fibers: Opportunities and Challenges. Adv. Mater..

[8] Park J., Lee K.H. (2012). Carbon Nanotube Yarns. Korean J. Chem. Eng..

[9] Fallah Gilvaei A., Hirahara K., Nakayama Y. (2011). In-Situ Study of the Carbon Nanotube Yarn Drawing Process. Carbon.

[10] Zhang D., Miao M., Niu H., Wei Z. (2014). Core-Spun Carbon Nanotube Yarn Supercapacitors for Wearable Electronic Textiles. ACS Nano.

[11] Im J., Jeong Y.H., Kim M.C., Oh D., Son J., Hyun K., Jeong B., Hong S., Lee J. (2024). Wet Spinning of Multi-Walled Carbon Nanotube Fibers. Carbon.

[12] Rabbani M.T., Sonker M., Ros A. (2020). Carbon Nanotube Dielectrophoresis: Theory and Applications. Electrophoresis.

[13] Tang J., Gao B., Geng H., Velev O.D., Qin L.C., Zhou O. (2003). Assembly of 1D Nanostructures into Sub-Micrometer Diameter Fibrils with Controlled and Variable Length by Dielectrophoresis. Adv. Mater..

[14] Plaado M., Mononen R.M., Lhmus R., Kink I., Saal K. (2011). Formation of Thick Dielectrophoretic Carbon Nanotube Fibers. Nanotechnology.

[15] Li W., Hennrich F., Flavel B.S., Dehm S., Kappes M., Krupke R. (2021). Principles of Carbon Nanotube Dielectrophoresis. Nano Res..

[16] Ismail I., Zubair A. Alignment of Interacting Multi-Wall Carbon Nanotubes in Suspension Using Dielectrophorectic Framework. Proceedings of the 12th International Conference on Electrical and Computer Engineering (ICECE).

[17] Jang Y., Kim S.M., Spinks G.M., Kim S.J. (2020). Carbon Nanotube Yarn for Fiber-Shaped Electrical Sensors, Actuators, and Energy Storage for Smart Systems. Adv. Mater..

[18] Foroughi J., Spinks G. (2019). Carbon Nanotube and Graphene Fiber Artificial Muscles. Nanoscale Adv..

[19] Presser V., Heon M., Gogotsi Y. (2011). Carbide-Derived Carbons—From Porous Networks to Nanotubes and Graphene. Adv. Funct. Mater..

[20] Plaado M., Kaasik F., Valner R., Lust E., Saar R., Saal K., Peikolainen A.-L., Aabloo A., Kiefer R. (2015). Electrochemical Actuation of Multiwall Carbon Nanotube Fiber with Embedded Carbide-Derived Carbon Particles. Carbon.

[21] Kiefer R., Plaado M., Harjo M., Tamm T. (2022). Tuning the Linear Actuation of Multiwall Carbon Nanotube Fibers with Carbide-Derived Carbon. Synth. Met..

[22] Kosidlo U., Omastova M., Micusik M., Ciric-Marjanovic G., Randriamahazaka H., Wallmersperger T., Aabloo A., Kolaric I., Bauernhansl T. (2013). Nanocarbon Based Ionic Actuators-a Review. Smart Mater. Struct..

[23] Baughman R.H., Cui C., Zakhidov A.A., Iqbal Z., Barisci J.N., Spinks G.M., Wallace G.G., Mazzoldi A., De Rossi D., Rinzler A.G. (1999). Carbon Nanotube Actuators. Science.

[24] Torop J., Arulepp M., Leis J., Punning A., Johanson U., Palmre V., Aabloo A. (2010). Nanoporous Carbide-Derived Carbon Material-Based Linear Actuators. Materials.

[25] Torop J., Arulepp M., Sugino T., Asaka K., Ja A., Lust E., Aabloo A. (2014). Microporous and Mesoporous Carbide-Derived Carbons for Strain Modification of Electromechanical Actuators. Langmuir.

[26] Kaasik F., Tamm T., Hantel M.M., Perre E., Aabloo A., Lust E., Bazant M.Z., Presser V. (2013). Anisometric Charge Dependent Swelling of Porous Carbon in an Ionic Liquid. Electrochem. Commun..

[27] Elhi F., Le Q.B., Kiefer R. (2025). Multifunctionality of Cellulose Multiwall Carbon Nanotube Composites in Polar Aprotic and Polar Protic Solvents. J. Appl. Polym. Sci..

[28] Khuyen N.Q., Elhi F., Le Q.B., Kiefer R. (2023). Sustainability of Multiwall Carbon Nanotube Fibers and Their Cellulose Composite. Sustainability.

[29] Harjo M., Tamm T., Anbarjafari G., Kiefer R. (2019). Hardware and Software Development for Isotonic Strain and Isometric Stress Measurements of Linear Ionic Actuators. Polymers.

[30] Kaempgen M., Chan C.K., Ma J., Cui Y., Gruner G. (2009). Printable Thin Film Supercapacitors Using Single-Walled Carbon Nanotubes. Nano Lett..

[31] Dehghanpour H., Yilmaz K. (2020). The Relationship between Resistances Measured by Two-Probe, Wenner Probe and C1760-12 ASTM Methods in Electrically Conductive Concretes. SN Appl. Sci..

[32] Schneider C., Rasband W., Eliceiri K. (2012). NIH Image to ImageJ: 25 Years of Image Analysis. Nat. Methods.

[33] Ma J., Tang J., Zhang H., Shinya N., Qin L.-C. (2009). Ultrathin Carbon Nanotube Fibrils of High Electrochemical Capacitance. ACS Nano.

[34] Kiefer R., Elhi F., Puust L., Peikolainen A.-L., Tamm T. (2022). Dual Function Composite Fibers of Cellulose with Activated Carbon Aerogel and Carbide Derived Carbon. J. Appl. Polym. Sci..

[35] Kiefer R., Aydemir N., Torop J., Tamm T., Temmer R., Travas-Sejdic J., Must I., Kaasik F., Aabloo A. (2014). Carbide-Derived Carbon as Active Interlayer of Polypyrrole Tri-Layer Linear Actuator. Sens. Actuators B Chem..

[36] Baskaran D., Mays J.W., Bratcher M.S. (2005). Noncovalent and Nonspecific Molecular Interactions of Polymers with Multiwalled Carbon Nanotubes. Chem. Mater..

[37] Lilloja J., Kibena-Põldsepp E., Sarapuu A., Kikas A., Kisand V., Käärik M., Merisalu M., Treshchalov A., Leis J., Sammelselg V. (2020). Nitrogen-Doped Carbide-Derived Carbon/carbon Nanotube Composites as Cathode Catalysts for Anion Exchange Membrane Fuel Cell Application. Appl. Catal. B Environ..

[38] Praats R., Käärik M., Kikas A., Kisand V., Aruväli J., Paiste P., Merisalu M., Sarapuu A., Leis J., Sammelselg V. (2021). Electroreduction of Oxygen on Cobalt Phthalocyanine-Modified Carbide-Derived Carbon/carbon Nanotube Composite Catalysts. J. Solid State Electrochem..

[39] Tsentalovich D.E., Headrick R.J., Mirri F., Hao J., Behabtu N., Young C.C., Pasquali M. (2017). Influence of Carbon Nanotube Characteristics on Macroscopic Fiber Properties. ACS Appl. Mater. Interfaces.

[40] Ajayan P.M., Schadler L.S., Giannaris C., Rubio A. (2000). Single-Walled Carbon Nanotube-Polymer Composites: Strength and Weakness. Adv. Mater..

[41] Ramón-Azcón J., Ahadian S., Estili M., Liang X., Ostrovidov S., Kaji H., Shiku H., Ramalingam M., Nakajima K., Sakka Y. (2013). Dielectrophoretically Aligned Carbon Nanotubes to Control Electrical and Mechanical Properties of Hydrogels to Fabricate Contractile Muscle Myofibers. Adv. Mater..

[42] Elhi F., Puust L., Kiefer R., Tamm T. (2023). Electrolyte Contribution to the Multifunctional Response of Cellulose Carbon Nanotube Fibers. React. Funct. Polym..

[43] Chmiola J., Yushin G., Gogotsi Y., Portet C., Simon P., Taberna P.L. (2006). Anomalous Increase in Carbon at Pore Sizes Less than 1 Nanometer. Science.

[44] Otero T.F., Martinez J.G., Asaka K. (2016). Faradaic and Capacitive Components of the CNT Electrochemical Responses. Front. Mater..

[45] Lyon J.L., Stevenson K.J. (2007). Anomalous Electrochemical Dissolution and Passivation of Iron Growth Catalysts in Carbon Nanotubes. Langmuir.

[46] Valero L., Otero T.F., Martinez J.G., Martínez J.G. (2014). Exchanged Cations and Water during Reactions in Polypyrrole Macroions from Artificial Muscles. ChemPhysChem.

[47] Barisci J.N., Spinks G.M., Wallace G.G., Madden J.D., Baughman R.H. (2003). Increased Actuation Rate of Electromechanical Carbon Nanotube Actuators Using Potential Pulses with Resistance Compensation. Smart Mater. Struct..

[48] Hughes M., Spinks G.M. (2005). Multiwalled Carbon-Nanotube Actuators. Adv. Mater..

[49] Geier S.M., Mahrholz T., Wierach P., Sinapius M. (2018). Morphology- and Ion Size-Induced Actuation of Carbon Nanotube Architectures. Int. J. Smart Nano Mater..

[50] Riemenschneider J., Temmen H., Monner H.P. (2007). CNT Based Actuators: Experimental and Theoretical Investigation of the in-Plain Strain Generation. J. Nanosci. Nanotechnol..

[51] Trunzer T., Fraga-García P., Tschuschner M.P.A., Voltmer D., Berensmeier S. (2022). The Electrosorptive Response of a Carbon Nanotube Flow-through Electrode in Aqueous Systems. Chem. Eng. J..

[52] Zhao D., Liu R., Luo C., Guo Y., Hou C., Zhang Q., Li Y., Jia W., Wang H. (2021). Dielectrophoretic Assembly of Carbon Nanotube Chains in Aqueous Solution. Adv. Fiber Mater..

[53] Wen L., Li F., Cheng H.M. (2016). Carbon Nanotubes and Graphene for Flexible Electrochemical Energy Storage: From Materials to Devices. Adv. Mater..

[54] Pandit B., Dhakate S.R., Singh B.P., Sankapal B.R. (2017). Free-Standing Flexible MWCNTs Bucky Paper: Extremely Stable and Energy Efficient Supercapacitive Electrode. Electrochim. Acta.

[55] Centi G., Perathoner S. (2011). Carbon Nanotubes for Sustainable Energy Applications. ChemSusChem.

[56] Liu C., Yan X., Hu F., Gao G., Wu G., Yang X. (2018). Toward Superior Capacitive Energy Storage: Recent Advances in Pore Engineering for Dense Electrodes. Adv. Mater..

[57] Su J., Huang D. (2016). Coupling Transport of Water and Ions Through a Carbon Nanotube: The Role of Ionic Condition. J. Phys. Chem. C.

[58] Torop J., Palmre V., Arulepp M., Sugino T., Asaka K., Aabloo A. (2011). Flexible Supercapacitor-like Actuator with Carbide-Derived Carbon Electrodes. Carbon.

[59] Pohl M., Kurig H., Tallo I., Jänes A., Lust E. (2017). Novel Sol-Gel Synthesis Route of Carbide-Derived Carbon Composites for Very High Power Density Supercapacitors. Chem. Eng. J..

[60] Malmberg S., Arulepp M., Tarasova E., Vassiljeva V., Krasnou I., Krumme A. (2020). Electrochemical Evaluation of Directly Electrospun Carbide-Derived Carbon-Based Electrodes in Different Nonaqueous Electrolytes for Energy Storage Applications. J. Carbon Res..

[61] Sun X., Cai M., Chen L., Qiu Z., Liu Z. (2017). Electrodes of Carbonized MWCNT-Cellulose Paper for Supercapacitor. J. Nanopart. Res..

[62] Felhősi I., Keresztes Z., Marek T., Pajkossy T. (2020). Properties of Electrochemical Double-Layer Capacitors with Carbon-Nanotubes-on-Carbon-Fiber-Felt Electrodes. Electrochim. Acta.

[63] Dall’Agnese Y., Rozier P., Taberna P.L., Gogotsi Y., Simon P. (2016). Capacitance of Two-Dimensional Titanium Carbide (MXene) and MXene/carbon Nanotube Composites in Organic Electrolytes. J. Power Sources.

[64] Borenstien A., Noked M., Okashy S., Aurbach D. (2013). Composite Carbon Nano-Tubes (CNT)/Activated Carbon Electrodes for Non-Aqueous Super Capacitors Using Organic Electrolyte Solutions. J. Electrochem. Soc..

[65] Lota K., Khomenko V., Frackowiak E. (2004). Capacitance Properties of poly(3,4-Ethylenedioxythiophene)/carbon Nanotubes Composites. J. Phys. Chem. Solids.

[66] Chen S., Qiu L., Cheng H.M. (2020). Carbon-Based Fibers for Advanced Electrochemical Energy Storage Devices. Chem. Rev..

[67] Foroughi J., Spinks G.M., Ghorbani S.R., Kozlov M.E., Safaei F., Peleckis G., Wallace G.G., Baughman R.H. (2012). Preparation and Characterization of Hybrid Conducting Polymer-Carbon Nanotube Yarn. Nanoscale.

[68] Hou Y., Ofori E.A., Gbologah L., Xiong Y., Mensah-darkwa K., Tawiah B., Fei B., Zhao X. (2025). Electrochemical Fiber Electrode Fabrication by Spinning: State-of- the-Art and Perspectives. ACS Electrochem..

[69] Hyeon J.S., Park J.W., Baughman R.H., Kim S.J. (2019). Electrochemical Graphene/carbon Nanotube Yarn Artificial Muscles. Sens. Actuators B Chem..

[70] Choi C., Kim K.M., Kim K.J., Lepró X., Spinks G.M., Baughman R.H., Kim S.J. (2016). Improvement of System Capacitance via Weavable Superelastic Biscrolled Yarn Supercapacitors. Nat. Commun..

[71] Zhai S., Jiang W., Wei L., Karahan H.E., Yuan Y., Ng A.K., Chen Y. (2015). All-Carbon Solid-State Yarn Supercapacitors from Activated Carbon and Carbon Fibers for Smart Textiles. Mater. Horiz..

[72] Ren J., Li L., Chen C., Chen X., Cai Z., Qiu L., Wang Y., Zhu X., Peng H. (2013). Twisting Carbon Nanotube Fibers for Both Wire-Shaped Micro-Supercapacitor and Micro-Battery. Adv. Mater..

