# Supplementary material for: Linear Actuation of Dielectrophoretic Formed Multi-Walled Carbon Nanotube Fiber with Carbide-Derived Carbon in Polar Aprotic and Polar Protic Solvents
Source: Materials (Basel). 2025 Jul 10;18(14):3254. doi: 10.3390/ma18143254 (PMC12300003; doi:10.3390/ma18143254)
Supplement: Supplementary file 1 [file materials-18-03254-s001.zip › materials-3727594-supplementary.pdf]

Supplementary

Linear Actuation of Dielectrophoretic Formed Multi-Walled Carbon Nanotube Fiber with Carbide-Derived Carbon in Polar Aprotic and Polar Protic Solvents

Chau B. Tran [1] , Quoc Bao Le [2,3] and Rudolf Kiefer [2,*]

[1] Faculty of Applied Sciences, Ton Duc Thang University, Ho Chi Minh City 700000, Vietnam; tranboichau@tdtu.edu.vn

[2] Conducting Polymers in Composites and Applications Research Group, Faculty of Applied Sciences, Ton Duc Thang University, Ho Chi Minh City 700000, Vietnam; qle@pittstate.edu

[3] National Institute for Materials Advancement, Pittsburg State University, Pittsburg, KS 66762, USA

* Correspondence: rudolf.kiefer@tdtu.edu.vn; Tel.: +84-784566419

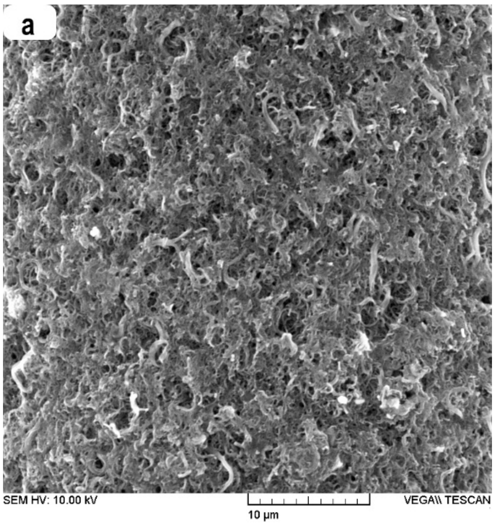

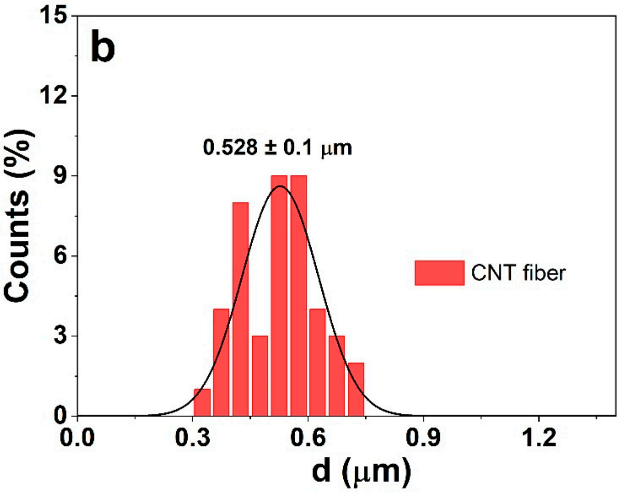

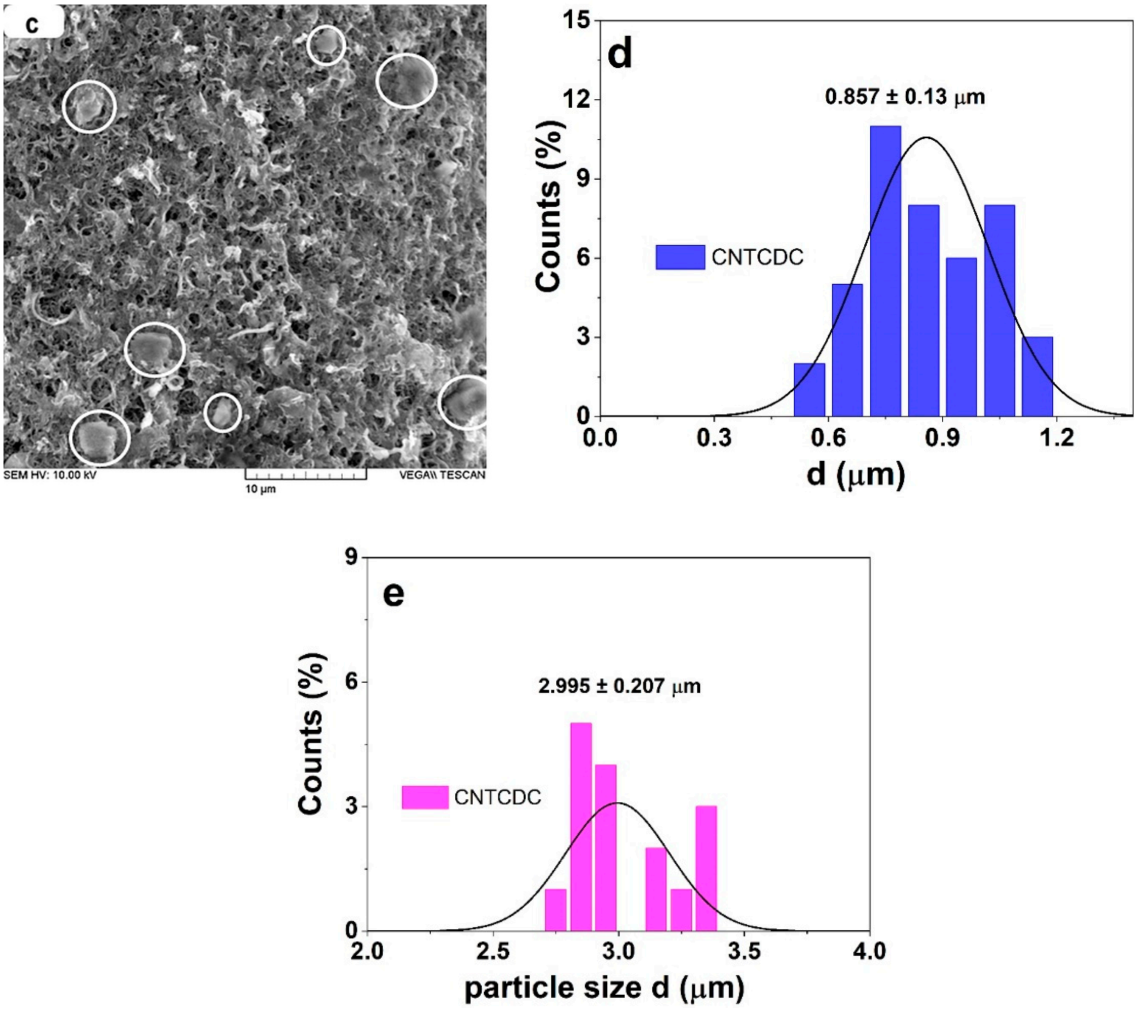

Figure S1. Analyse using ImageJ software on SEM images (scale bar 10 μm) showing the surface of CNT fiber in a). The average pore size analysis (diameter of pores, d) of CNT fiber is presented in b). The SEM surface image of CNTCDC is displayed in c) and the circles showing the CDC particles, with the diameter of pores shown in d). The CDC's particle size (showing diameter d) in CNT is presented in e).

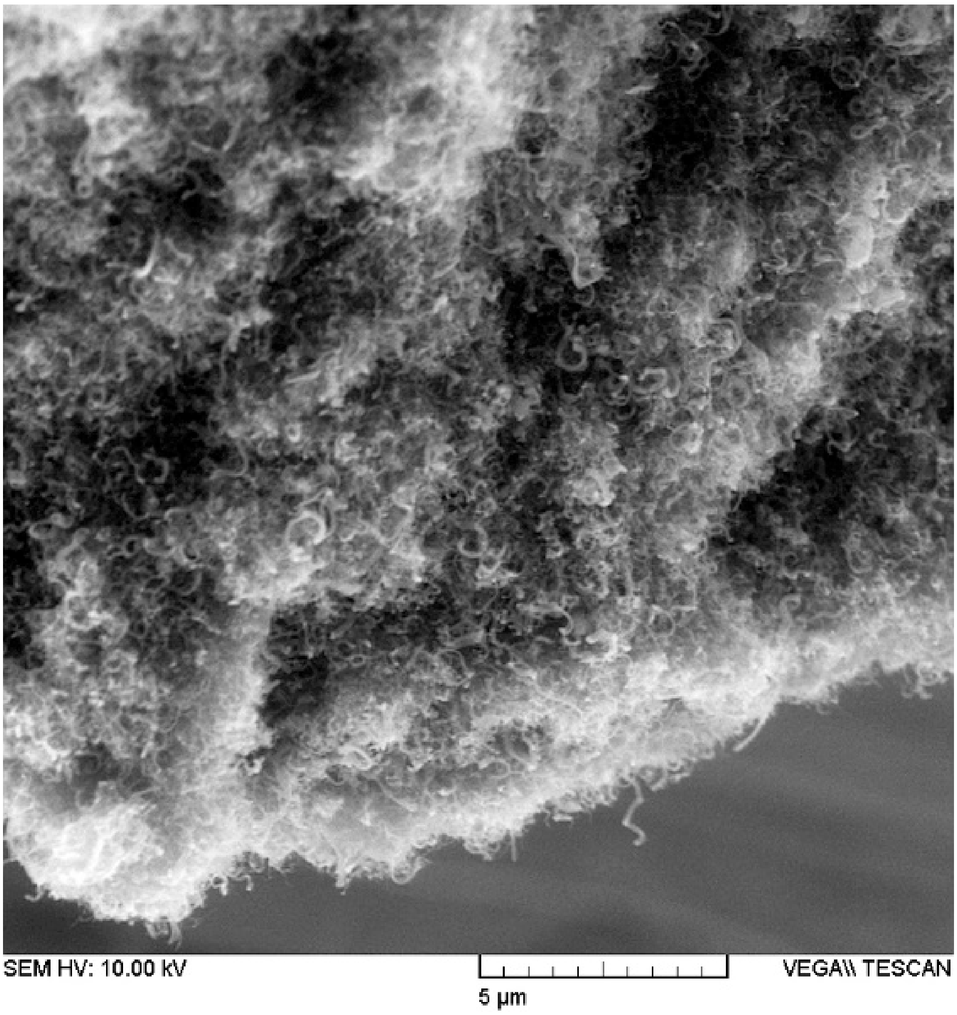

Figure S2. SEM image (scale bar 5 µm) of CNT fiber higher cross-section resolution with average CNT length at the range of 1 -2 µm.

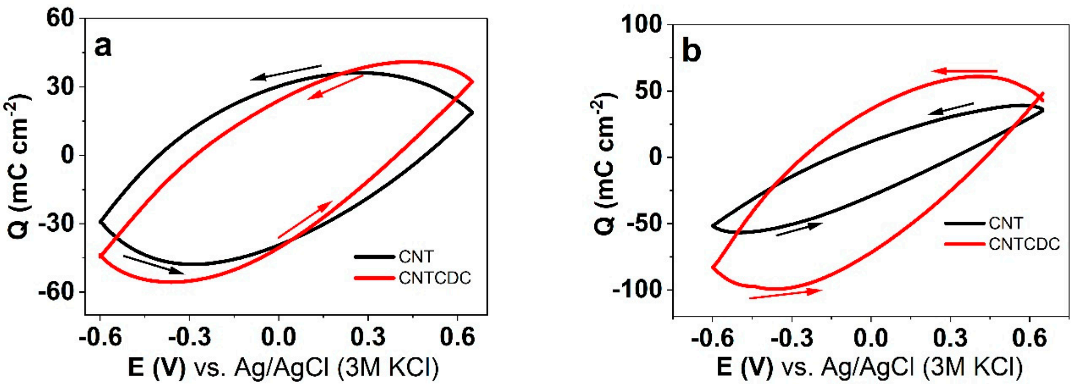

Figure S3. Coulovoltametry of 3rd cycle (charge density Q against potential E) of CNT fiber (black line) and CNTCDC fiber (red line) measurements in LiTFSI-PC presented in a) and LiTFSI-aq in b). The arrows show the direction of the scans.

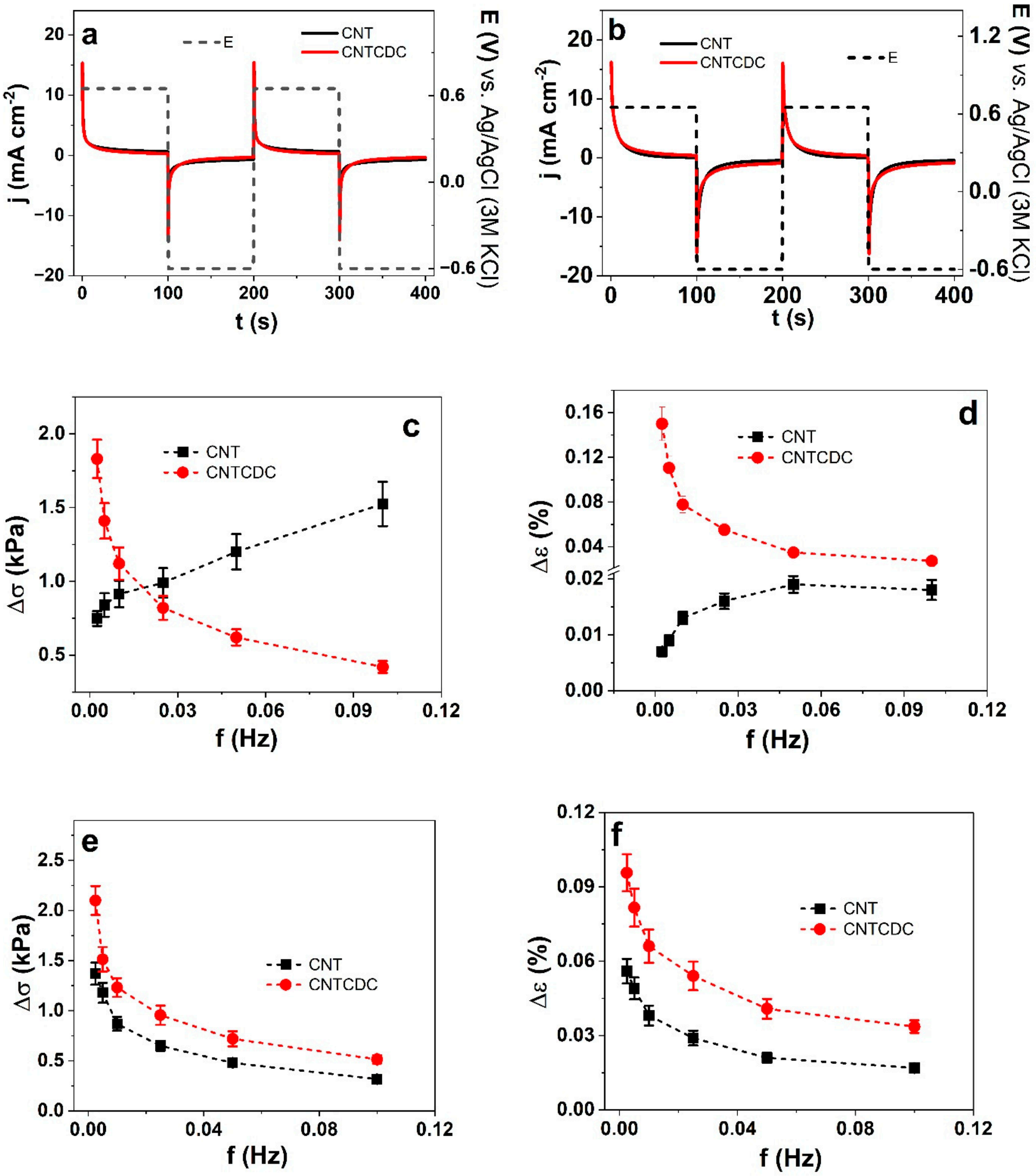

Figure S4. EMD chronoamperometric measurements of CNT and CNTCDC fiber of current density time curves against time (cycle 3rd to 4th) are presented for LiTFSI-PC in a) and LiTFSI-aq in b). The stress and strain difference in LiTFSI-PC of CNT and CNTCDC fiber against the

applied frequencies f are shown in c) and d). The stress and strain differences in LiTFSI-aq against frequencies are presented in e) and f).

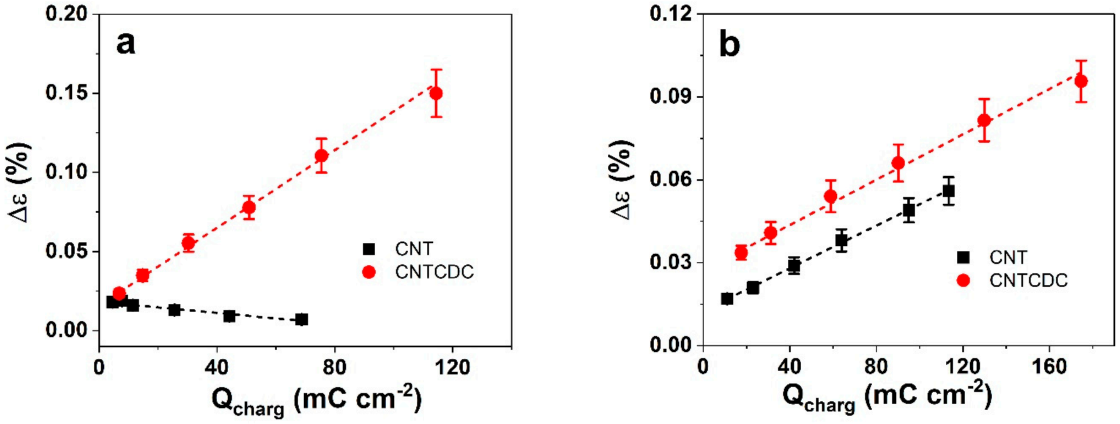

Figure S5. Square wave steps combined with EMD measurements of CNT (black line, ■) and CNTCDC fiber (red line, ●) at potential range E (0.65V to -0.6V, dashed line) showing in a) strain differences Δε against charge density Q_{charg} in LiTFSI-PC and in b) strain differences Δε in LiTFSI-aq against charge density in b). The dashed lines represent the linear fit, showing for orientation only.

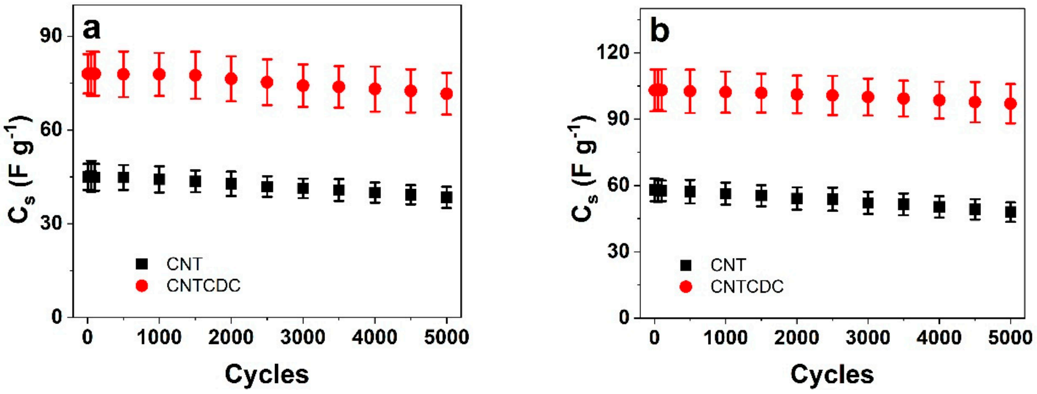

Figure S6. Long-term chronopotentiometry measurements showing specific capacitance Cs against cycles (5000 cycles) of CNT (■) at 32 A g^{-1} and CNTCDC (●) fiber at 34 A g^{-1} with those shown in polar aprotic solvent (PC) in a) and polar protic (Aq) in b).

Table S1. Comparison of carbon nanotubes (CNT) and carbide-derived carbon (CDC) composites in fiber actuation and their specific capacitance C_s, capacitance retention (CR) in organic and aqueous electrolytes

Material	Electrolytes	Actuation	C_s	CR (%)
MWCNT/MnO_2 twisted two fibers[1]	$LiPF_6$ (EC, DC, EMC) 0 – 1V	NA	15.6 F/g	1000 cycles, 10 V/s, 98% CR
Graphene CNT yarn[2]	0.2M $TBAPF_6$ in PC, 0 to -1V	-0.05% linear strain	22.5 F/g (100 mV/s)	NA
Birolled CNT yarn with 90% MnO_2[3]	PVA/LiCl gel	NA	888 mF/cm^2 (2 mA/cm^2)	1000 cycle, 100 mV/s, 98.4% CR
Twist spun MWCNT yarn[4]	0.5M $TBAPF_6$ in ACN, ± 2.5V	0.5% linear strain at -2.5V (mixed actuation)	26 F/g (1V/s)	NA
Activated carbon and carbon yarn[5]	PVA/H_3PO_4 gel 1.0V to 0.0V	NA	10 cm length, 82.1 F/g (2 mV/s)	10.000 cycles (20 mV/s), 86.6% CR
Electro spun CDC fiber[6]	1.5M EMIm-TFSI in ACN, 0.0V to 3.0V	NA	105.6 F/g (0.5 mA/cm^2)	3000 cycles (20 mV/s) 100% CR
DEP formed CNTCDC (10 wt.%) fiber (our work)	0.1M LiTFSI in PC, 0.65V to -0.6V	0.1% linear strain, expansion at -0.6V	170.3 ± 13 F/g (0.8 A/g)	5000 cycles (32 A/g) 91.7% CR
	0.1M LiTFSI in aq 0.65V to -0.6V	0.047% linear strain at charging (0.65V)	223 ± 17 F/g (0.8 A/g)	5000 cycles (32 A/g) 94.2% CR

Abbreviations: NA not available, $LiPF_6$ (Lithium hexafluorophosphate), EC (Ethylene carbonate), DC (diethyl carbonate), EMC (ethyl methyl carbonate), $TBAPF_6$ (tetrabutylammonium hexafluorophosphate), LiCl (lithium chloride), PVA (polyvinylalcohol), ACN (acetonitrile), H_3PO_4 (phosphoric acid), EMIm-TFSI (1-Ethyl-3-methylimidazoliumbis(trifluormethylsulfonyl)amid)

References

1. Ren, J.; Li, L.; Chen, C.; Chen, X.; Cai, Z.; Qiu, L.; Wang, Y.; Zhu, X.; Peng, H. Twisting Carbon Nanotube Fibers for Both Wire-Shaped Micro-Supercapacitor and Micro-Battery. *Adv. Mater.* **2013**, *25*, 1155–1159, doi:10.1002/adma.201203445.
2. Hyeon, J.S.; Park, J.W.; Baughman, R.H.; Kim, S.J. Electrochemical Graphene/carbon Nanotube Yarn Artificial Muscles. *Sensors Actuators, B Chem.* **2019**, *286*, 237–242, doi:10.1016/j.snb.2019.01.140.
3. Choi, C.; Kim, K.M.; Kim, K.J.; Lepró, X.; Spinks, G.M.; Baughman, R.H.; Kim, S.J. Improvement of System Capacitance via Weavable Superelastic Biscrolled Yarn Supercapacitors. *Nat. Commun.* **2016**, *7*, 1–8, doi:10.1038/ncomms13811.
4. Mirfakhrai, T.; Oh, J.; Kozlov, M.; Fok, E.C.W.; Zhang, M.; Fang, S.; Baughman, R.H.; Madden, J.D.W. Electrochemical Actuation of Carbon Nanotube Yarns. *Smart Mater. Struct.* **2007**, *16*, S243–S249, doi:10.1088/0964-1726/16/2/S07.
5. Zhai, S.; Jiang, W.; Wei, L.; Karahan, H.E.; Yuan, Y.; Ng, A.K.; Chen, Y. All-Carbon Solid-State Yarn Supercapacitors from Activated Carbon and Carbon Fibers for Smart Textiles. *Mater. Horizons* **2015**, *2*, 598–605, doi:10.1039/C5MH00108K.
6. Malmberg, S.; Arulepp, M.; Tarasova, E.; Vassiljeva, V.; Krasnou, I.; Krumme, A. Electrochemical Evaluation of Directly Electrospun Carbide-Derived Carbon-Based Electrodes in Different Nonaqueous Electrolytes for Energy Storage Applications. *J. Carbon Res.* **2020**, *6*, 59, doi:10.3390/c6040059.
